# Diversity and Distribution of Non-Reducing Polyketide Synthases (NR-PKSs) in Ascomycota (Fungi)

**DOI:** 10.3390/jof11090641

**Published:** 2025-08-29

**Authors:** Pritam Chattopadhyay, Goutam Banerjee

**Affiliations:** 1 Department of Botany, M.U.C. Women’s College, University of Burdwan, Bardhaman 713104, West Bengal, India; pritamchattopadhyay@mucwcburdwan.org; 2 Department of Food Science and Human Nutrition, University of Illinois at Urbana-Champaign, Urbana, IL 61801, USA

**Keywords:** NR-PKS diversity, Ascomycota, phylogeny, biosynthetic gene cluster (BGC), signature domains, protein modeling

## Abstract

(1) Background: This study highlights the diversity and distribution of non-reducing polyketide synthases (NR-PKSs) in Ascomycota and their role in producing bioactive aromatic polyketides. (2) Methods: A reference dataset of non-NR-PKSs was compiled from published literature and cross-examined using NaPDoS2 and Kyoto Encyclopedia of Genes and Genomes Ortholog (KEGG KO) databases. Signature domains were validated through Pfam and CDD, while phylogenetic classification was conducted by comparing the dataset with the NaPDoS2 reference tree. Cluster support was derived from KEGG KO and homology-based modeling. Additionally, NR-PKS clade distribution across KEGG genomes was analyzed, and co-expression patterns were examined using STRING. (3) Results: This study identified nine distinct clades of NR-PKSs, six of which are supported by unique KEGG Orthology (KO) numbers. These clades are as follows: clade 1: polyketide synthase A (PksA, K15316); clade 2: fusarubinsynthase 1 (Fsr1); clade 3: white A (WA, K15321); clade 4: polyketide synthase citrinin (PksCT); clade 5: zearalenone synthase 1 (Zea1, K15417); clade 6: orsellinic acid synthase A (OrsA, K15416); clade 7: aurofusarin polyketide synthase A (AptA, K15317); clade 8: monodictyphenone polyketide synthase G (MdpG, K15415); and clade 9: bikaverin polyketide synthase (Bik1). The present investigation also reports incongruency in the distribution of different NR-PKSs and fungi phylogeny within the phylum Ascomycota. (4) Conclusions: The distribution of NR-PKSs in Ascomycota defies phylogenetic boundaries, reflecting the impact of horizontal gene transfer, gene loss, and ecological adaptation.

## 1. Introduction

Polyketides are structurally diverse secondary metabolites produced by microorganisms, plants, and fungi, many of which exhibit antibiotic, antifungal, anticancer, and immunosuppressive properties [1,2]. In fungi, particularly in Ascomycota, their biosynthesis is facilitated by polyketide synthase (PKS) enzymes [3]. Among the PKS classes, iterative type I non-reducing PKSs (NR-PKSs) are essential for synthesizing aromatic polyketides, which play crucial ecological and pathogenic roles [4]. NR-PKSs are responsible for the biosynthesis of a wide array of aromatic secondary metabolites, many of which possess potent biological activities. For example, mycotoxins such as aflatoxins and sterigmatocystins produced by *Aspergillus flavus* and *Aspergillus nidulans* are carcinogenic polyketides synthesized by NR-PKSs like PksA [5]. Protective pigments like melanin are synthesized by Alb1 in *Aspergillus fumigatus*, contributing to virulence and resistance to environmental stress [6]. Bioactive pigments and cytotoxic compounds, including anthraquinones and azaphilones from *Fusarium* and *Penicillium*, exhibit antitumor and antimicrobial activities [7]. The pks1 gene encodes an NR-PKS involved in anthraquinones biosynthesis in *Monascus purpureus* [8]. Notably, azaphilone pigments are multifunctional molecules with significant potential applications in the food, pharmaceutical, and textile industries among others [9]. Consequently, the metabolic versatility of NR-PKSs has made them attractive targets for bioengineering and heterologous expression to facilitate the discovery of novel compounds [7].

NR-PKSs are a distinct class of fungal enzymes that synthesize aromatic polyketides without reductive modifications. Their domain architecture governs product specificity, cyclization patterns, and release mechanisms. Their core domain architecture typically includes a starter unit acyl carrier protein transacylase (SAT), ketosynthase (KS), acyltransferase (AT), product template (PT), acyl carrier protein (ACP), and often a thioesterase (TE) domain [10]. Unlike reducing PKSs, NR-PKSs lack reductive domains such as ketoreductase (KR), dehydratase (DH), and enoylreductase (ER), resulting in the formation of highly reactive poly-β-ketone intermediates that spontaneously cyclize into aromatic compounds [1,11]. These enzymes are particularly abundant in Ascomycota, where they are involved in the production of pigments, toxins, and other bioactive secondary metabolites. The modular architecture of NR-PKSs allows for the generation of diverse chemical scaffolds through variations in cyclization patterns and polyketide chain lengths [7]. Among the domains, the PT domain plays a critical role in determining the regioselectivity of aromatic ring formation—a key distinguishing feature from other PKS classes. Additionally, NR-PKSs often function in concert with various tailoring enzymes, including oxidases, methyltransferases, and glycosyltransferases, further expanding the structural and functional diversity of their metabolic products [11].

NR-PKSs are widely distributed among filamentous Ascomycota, including *Aspergillus*, *Penicillium*, *Fusarium*, and *Colletotrichum* species. Throckmorton et al. (2015) reported that the NR-PKS repertoire in filamentous fungi emerged from an ancestral gene duplication burst, followed by lineage-specific losses, horizontal gene transfer (HGT), and gradual functional innovation [12]. Genomic studies have shown that individual Ascomycete genomes can encode up to 20 or more polyketide synthase (PKS) genes, with a significant proportion belonging to the NR-PKS class [4]. The evolutionary history of NR-PKS genes reflects a combination of vertical descent and horizontal gene transfer (HGT), resulting in a mosaic distribution of PKS gene clusters across different taxa [13]. For example, the conservation of aflatoxin biosynthetic genes among *Aspergillus* species suggests ancient lineage-specific retention, while the presence of unique PKS clusters in endophytic fungi points to more recent HGT events [14]. Additionally, the expansion of NR-PKS genes is often linked to ecological niche adaptation. Pathogenic fungi typically exhibit a greater diversity of NR-PKSs that contribute to host–pathogen interactions, whereas saprophytic species utilize these enzymes for chemical defense and competition within their environments [1].

Recent advances in genomics and synthetic biology are increasingly unlocking their potential for drug discovery and industrial applications. Key catalytic domains, particularly the product template (PT) and thioesterase (TE) domains, have been repurposed to engineer new biosynthetic pathways for the production of artificial polyketides [11]. Techniques such as CRISPR-based genome editing and modular expression systems have enabled the manipulation of NR-PKS gene clusters, resulting in the generation of diverse metabolite libraries [7]. For instance, CRISPR-mediated knockout of pksA abolishes aflatoxin production, offering promising applications in food safety [15]. Additionally, advanced structural prediction tools like AlphaFold2 are being employed to model NR-PKS architectures and inform rational enzyme engineering strategies [16]. The creation of hybrid NR-PKSs—by combining PT and TE domains from different fungal sources—has further facilitated the design of unnatural biosynthetic pathways capable of producing novel compounds with enhanced bioactivity or reduced toxicity [17]. Despite these advances, there remains a significant demand for the discovery of novel iterative type I NR-PKSs to meet current industrial and pharmaceutical needs. This study reports the distribution and diversity of NR-PKSs in Ascomycete fungi, offering valuable insights for applications in the pharmaceutical and food industries.

## 2. Materials and Methods

### 2.1. Data Retrieval

**Figure 1:**
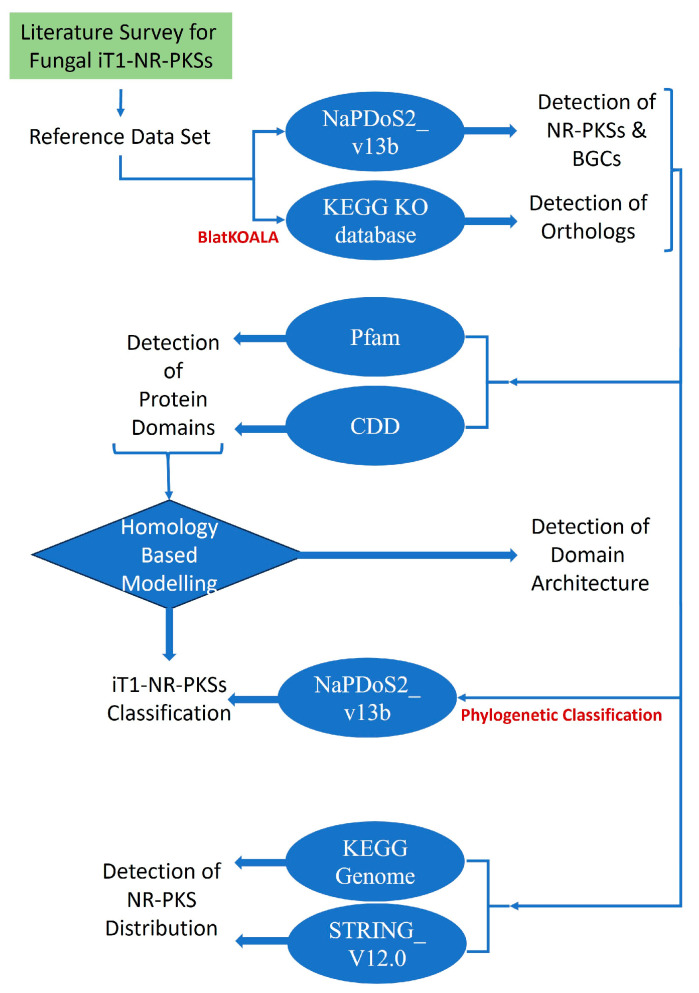
Detailedstep-by-step analysis workflow.

A reference dataset of reported NR-PKS genes and proteins was compiled from published literature (Table S1). The corresponding UniProt or NCBI Protein IDs for these proteins were retrieved from their respective databases (Table S1). Protein sequences in FASTA format were obtained using these IDs from either the UniProt or NCBI Protein database (Table S2). The compiled FASTA file was subsequently submitted to KEGG BlastKOALA (https://www.kegg.jp/blastkoala/ last accessed on 31 March 2025) to identify orthologous groups and assign KEGG Orthology (KO) numbers. To confirm the functional nature of the identified PKS orthologs, the sequences were further analyzed using Natural Product Domain Seeker 2 (NaPDoS2_v13b; https://npdomainseeker.sdsc.edu/napdos2 last accessed on 31 March 2025) [18]. The overall analysis workflow employed in this study is illustrated in Figure 1.

### 2.2. Screening for NR-PKSs

Initially, NaPDoS2_v13b was used to confirm the identity and nature of the PKS orthologs using default search parameters: domain type set to ketosynthase (KS), comparison type as protein, input sequence type as protein, BLAST program as blastp, minimum match length of 200, and an e-value cutoff of 1 × 10^−8^. To identify the presence of functional domains such as starter unit acyltransferase (SAT), ketosynthase (KS), acyltransferase (AT), product template (PT), acyl carrier protein (ACP), thioesterase (TE), ketoreductase (KR), dehydratase (DH), and enoylreductase (ER) within the PKS protein orthologs, a combination of bioinformatics tools and domain annotation databases was employed (Table S3). The Protein family database (Pfam) was accessed through InterPro (https://www.ebi.ac.uk/interpro/result/InterProScan last accessed on 31 March 2025) to scan protein sequences for known conserved domains [19]. The presence or absence of these domains was further validated using the Conserved Domain Database (CDD) at NCBI (https://www.ncbi.nlm.nih.gov/Structure/cdd/wrpsb.cgi last accessed on 31 March 2025) [20].

### 2.3. Phylogenetic Classification of NR-PKSs

To investigate different types of iterative type I NR-PKSs, a phylogeny-based classification scheme was employed. NaPDoS2 identifies ketosynthase (KS) domains and classifies them using a robust phylogenetic framework that integrates well-established biosynthetic knowledge and characterized PKS functions. The domain classification scheme used by NaPDoS2 is derived from KS sequence phylogenies and their relationships to the known biochemical roles, gene architectures, and structural features of the associated metabolites. This classification also considers the genomic context of KS domains found across diverse genomic and metagenomic datasets and is aligned with annotations generated by antiSMASH 6.0 [18]. Accordingly, in the present study, FASTA-formatted sequences of all identified iterative type I NR-PKSs were analyzed by querying them against the NaPDoS2 reference tree, which contains 414 representative sequences encompassing all known PKS classes and subclasses. The closest phylogenetic matches for each sequence were identified and recorded.

### 2.4. Homology Modeling and Evaluation of the Tertiary Structure

For further investigation of different classes of iterative type I NR-PKSs, the 3D structures of selected proteins were predicted using the SWISS-MODEL server. Initially, structural templates were identified through the ExPASy web portal (https://swissmodel.expasy.org/, last accessed on 31 March 2025), and the target sequences were aligned to these templates. The SWISS-MODEL server, incorporating the AlphaFold v2 method, was then used to construct the protein models and conduct preliminary quality assessments. To ensure the accuracy and reliability of the predicted 3D structures, the models were further evaluated using a combination of analytical tools, including MolProbity [21], and Ramachandran plot [22] analysis.

### 2.5. Distribution of NR-PKSs in Ascomycota

Due to the high degree of sequence similarity among various classes of polyketide synthases (PKSs), simple BLAST-based approaches can yield false-positive results. To investigate the global distribution of different types of iterative type I NR-PKSs within the Ascomycota group, two complementary strategies were employed. First, we analyzed the co-expression of multiple type I NR-PKSs using protein–protein interaction (PPI) networks derived from the STRING database (version 12.0), covering the entire Ascomycota clade [23]. In the second approach, KO numbers and/or enzyme names were used to accurately identify the corresponding proteins from fully annotated genomes. Although this method is labor-intensive, it offers a higher level of confidence in gene identification. To identify overlaps and the shared presence of interactive type I NR-PKSs across genomes, Venn diagrams were generated to visualize the distribution of common genes.

## 3. Results

### 3.1. Identification of Fungal NR-PKSs

Based on experimental records, an initial set of 20 fungal NR-PKS candidates were selected as query sequences (Table S1). Of the 20 candidates, 17 were confirmed as type I NR-PKSs; associated BGCs and orthologs were also identified (Table 1). Notably, both WA (Q03149) and PksP (Q4WZA8) were mapped to the same BGC product and KO number, indicating that they represent the same NR-PKS type. Several of the identified NR-PKSs were also associated with common KO numbers. For instance, K15317 (APTA; asperthecin synthase) was linked to four distinct BGCs: alternariol, asperthecin, TAN-1612, and viridicatumtoxin, highlighting functional redundancy among fungal NR-PKSs (Table 1).

### 3.2. Identification of the Protein Domains for NR-PKSs

**Figure 2:**
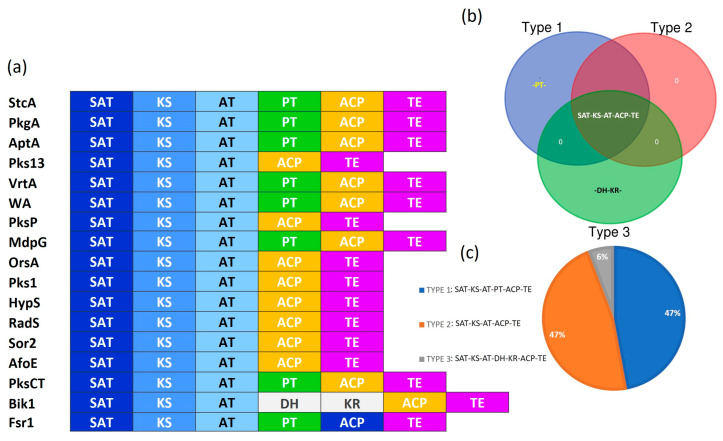
Domain architecture of the NR-PKSs: (**a**) bar diagram showing distribution of non-reducing and reducing domains across 17 NR-PKSs; (**b**) Venn diagram representing three types of iT1-NR-PKSs (TYPE 1: SAT-KS-AT-PT-ACP-TE; TYPE 2: SAT-KS-AT-ACP-TE; and TYPE 3: SAT-KS-AT-DH-KR-ACP-TE); (**c**) pie chart showing distribution percentage of these three types of NR-PKSs.

Six Pfam IDs were retrieved for the domains commonly present in interactive type 1 non-reducing polyketide synthases (iT1-NR-PKSs): SAT (PF16073), KS (PF00109), AT (PF00698), PT (PF00550), and ACP (PF00975). Additionally, Pfam IDs were also retrieved for three reductive domains to eliminate any false-positive results: TE (PF08659), DH (PF08242), and ER (PF00106) (Table S4). The distribution of domains is presented in Figure 2a. Three different types of domain architecture were observed (Figure 2b). The results indicated that 47% of the iT1-NR-PKSs have a specific domain distribution (SAT-KS-AT-PT-ACP-TE) pattern (Figure 2c). However, in another 47% of the iT1-NR-PKS, PT domains were not observed (Figure 2c). Among the 17 iT1-NR-PKSs, only one (Bik1) may contain DH and KR domains (Figure 2c).

### 3.3. Classification of Fungal NR-PKSs

**Figure 3:**
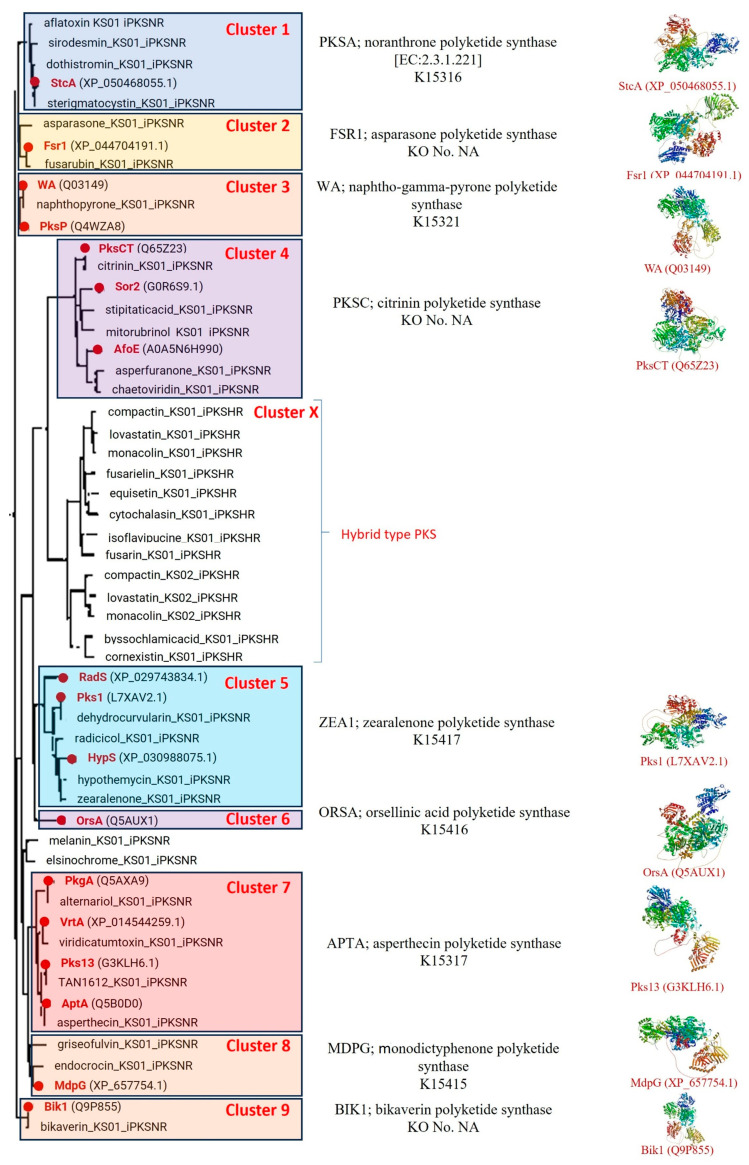
Phylogenetic tree showing nine distinct NR-PKS clusters and one interactive hybrid PKS group. Most of the clustersare supported by a unique KEGG ortholog (KO) number and associated with a specific biosynthetic gene cluster (BGC) product. Representative 3D protein models from each of the nine NR-PKS clusters are also included.

In this study, 17 NR-PKSs were mapped against a reference phylogenetic tree in NaPDoS2 containing 414 sequences covering all class and subclass assignments, and the closest matches are shown in Figure 3. The 17 iterative type I NR-PKSs (iT1-NR-PKSs) represent nine of these clusters (Clusters 1–9), while the tenth cluster corresponds to interactive hybrid-type PKSs, a related but distinct class evolutionarily derived from NR-PKSs. Most of the clusters (six out of nine) are supported by separate KO nos. and the distinct chemical nature of the BGCs (Figure 3). For instance, StcA represents Cluster 1 of the phylogenetic tree, and the chemical nature of the BGCs produced from this cluster is noranthrone (e.g., aflatoxin, sirodesmin, sterigmatocystin, etc.), supported by the ortholog identified from the KEGG database (K15316). Fsr1 represents Cluster 2, and the chemical nature of the BGCs produced from this cluster is asparasone (e.g., fusarubin). WA and PksP represent Cluster 3, supported by ortholog K15321, and the chemical nature of the BGCs produced from this cluster is naptho-gamma-pyrone (e.g., white A). PksCT, Sor2, and AfoE represent Cluster 4, responsible for aromatic polyphenolic compound synthesis (e.g., citrinin, stipitaticacid, asperfuranone, etc.). Cluster 5, represented by RadS, Pks1, and HypS, is supported by KO number K15417 and is associated with zearalenone-type BGCs, producing compounds like dehydrocurvularin, radicicol, and hypothemycin. Cluster 6, represented by OrsA, corresponds to orsellinic acid biosynthesis and is supported by KO K15416. Cluster 7 includes PkgA, VrtA, Pks13, and AptA, all linked to asperthecin-type BGCs (e.g., alternariol, viridicatumtoxin, TAN-1612), supported by KO K15317. Cluster 8, represented by MdpG, is associated with monodictyphenone-type compounds such as griseofulvin and endocrocin and is supported by KO K15415. Finally, Cluster 9 is represented solely by Bik1 and corresponds to the bikaverin biosynthetic pathway (Figure 3).

### 3.4. Tertiary Structures of NR-PKSs and Validation

**Figure 4:**
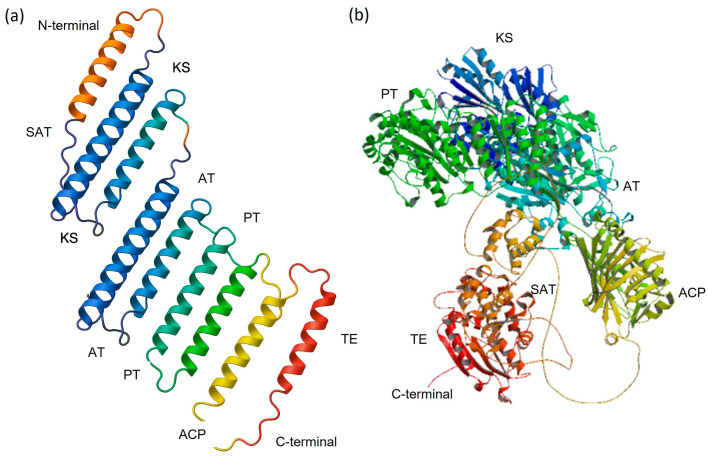
Tertiary (3D) structure of NR-PKSs: (**a**) schematic 3D presentation of iT1-NR-PKSs to locate the location of the domains SAT-KS-AT-PT-ACP-TE; (**b**) actual 3D structure and the location of the domains for WA (Q03149).

Protein 3D models were generated for one representative from each of the nine iT1-NR-PKS clusters identified in the phylogenetic tree (Figure 3). To visualize domain organization in the tertiary structure, a schematic of WA alongside its predicted 3D structure is shown in Figure 4. AlphaFold2 predictions revealed a moderately conserved architecture across all test proteins, characterized by a dimeric KS–AT core flanked by ACP and TE domains. Representative models include StcA (Cluster 1, Figure S1(Aa1)), Fsr1 (Cluster 2, Figure S1(Bb1)), WA (Cluster 3, Figure S1(Cc1)), PksCT (Cluster 4, Figure S1(Dd1)), Pks1 (Cluster 5, Figure S1(Ee1)), OrsA (Cluster 6, Figure S1(Ff1)), Pks13 (Cluster 7, Figure S1(Gg1)), MdpG (Cluster 8, Figure S1(Hh1)), and Bik1 (Cluster 9, Figure S1(Ii1)).

In template-based homology modeling, selecting an appropriate template is critical and is primarily guided by sequence similarity and identity coverage (Table S4). In this study, all chosen templates showed ≥60% sequence similarity (except Fsr1 at 57%) and near-complete sequence identity coverage (100% for most, except Fsr1 at 84.98% and Bik1 at 99.70%). All experimental models exhibited a GMQE score above 0.8, indicating high-quality predictions. Structural validations using MolProbity and Ramachandran plot analysis confirmed model reliability. All models had MolProbity and Clash scores below 2.0 and exhibited over 90% residues in Ramachandran favored regions (Table S4). Individual Ramachandran plots for each test protein are provided: StcA (Figure S1(Aa2)), Fsr1 (Figure S1(Bb2)), WA (Figure S1(Cc2)), PksCT (Figure S1(Dd2)), Pks1 (Figure S1(Ee2)), OrsA (Figure S1(Ff2)), Pks13 (Figure S1(Gg2)), MdpG (Figure S1(Hh2)), and Bik1 (Figure S1(Ii2)).

KS (ketosynthase) domains across NR-PKSs are highly conserved, characterized by a catalytic Cys-His-His triad and conserved motifs such as DTACSSSL, EXHGTGTXXGDP, and GSXKXNXGHXE, as identified through homology modeling and phylogenetic analysis. Structural models reveal variability in active-site cavity volumes correlating with elongation cycle numbers: most NR-PKSs (e.g., WA, PksP, MdpG, Bik1) exhibit intermediate cavity sizes (~750 Å^3^), whereas others (e.g., PksCT, Sor2, AfoE) show larger volumes (~1400 Å^3^). The PT domain, notably in PksA, adopts a double hot-dog fold with a deep substrate-binding pocket, entry-regulating helices, and a His–Asp catalytic dyad. Among the nine experimental NR-PKSs, major differences were found in PT domain structures, correlating with distinct cyclization patterns (e.g., C2–C7, C4–C9, C6–C11). In iT1-NR-PKSs such as WA, PksCT, OrsA, MdpG, and Bik1, a TE domain (~250–300 aa) is fused at the C-terminus. Structural analysis of PksA’s TE domain reveals a characteristic lid loop, suggesting a conserved termination mechanism.

### 3.5. Distribution of NR-PKSs Among the Members of Ascomycota

**Figure 5:**
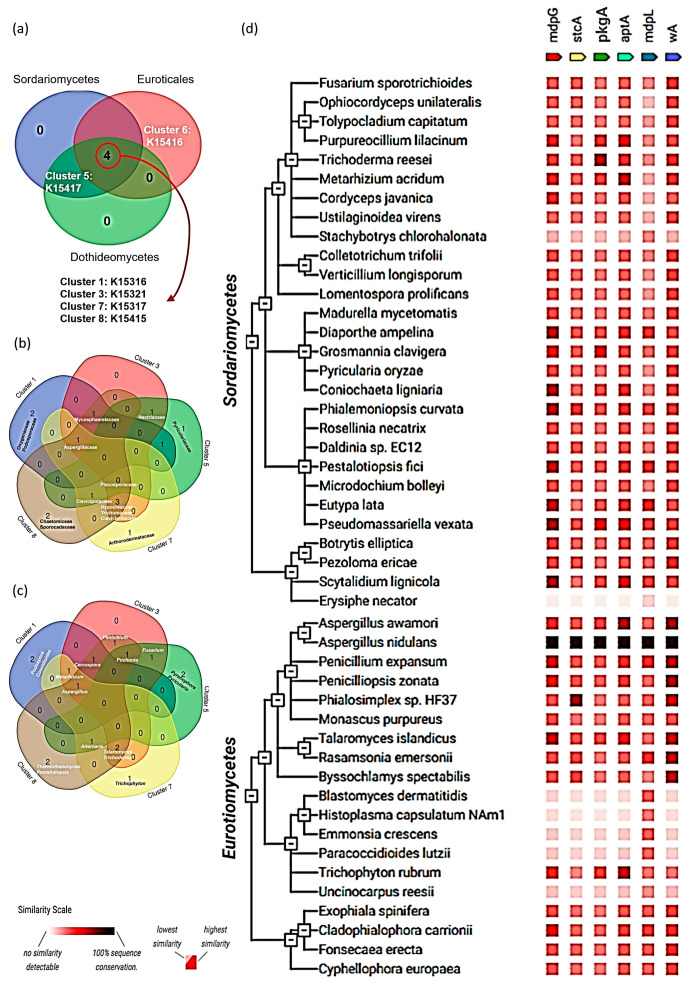
Distribution of different NR-PKSs across the fungi phylum Ascomycota: (**a**) distribution of noranthrone synthase (K15316), zearalenone synthase (K15417), orsellinic acid synthase (K15416), asperthecin synthase (K15317), and monodictyphenone synthase (K15415) across three classes (Sordariomycetes, Eurotiomycetes, and Dothideomycetes) of subphylum Pezizomycotina; (**b**) distribution of noranthrone synthase (K15316), zearalenone synthase (K15417), asperthecin synthase (K15317), and and monodictyphenone synthase (K15415) across families under the subphylum Pezizomycotina; (**c**) distribution of noranthrone synthase (K15316), zearalenone synthase (K15417), asperthecin synthase (K15317), and monodictyphenone synthase (K15415) across genus under the subphylum Pezizomycotina; (**d**) phylogenetic tree showing co-expression of wA, stcA, mdpG, aptA, mdpL, and orsA across the classes Sordariomycetes and Eurotiomycetes of subphylum Pezizomycotina.

In this study, six distinct types of NR-PKSs (each associated with a unique KO number) were identified across 40 species belonging to 16 genera, 14 families, 8 orders, and 3 classes within the subphylum Pezizomycotina using the KEGG genome database (Table S5). A Venn diagram (Figure 5a) illustrates the distribution of iT1-NR-PKSs among the three classes of Pezizomycotina: Sordariomycetes, Eurotiomycetes, and Dothideomycetes. Four NR-PKS types—Cluster 1 (noranthrone synthase, K15316), Cluster 3 (naphtho-γ-pyrone synthase, K15321), Cluster 7 (asperthecin synthase, K15317), and Cluster 8 (monodictyphenone synthase, K15415)—are shared across all three classes. Cluster 5 (zearalenone synthase, K15417) is restricted to Sordariomycetes and Dothideomycetes, while Cluster 6 (orsellinic acid synthase, K15416) is uniquely expressed in *Aspergillus nidulans* (family Aspergillaceae, order Eurotiales, class Eurotiomycetes). Further Venn analyses at the family (Figure 5b) and genus (Figure S2) levels reveal that Aspergillaceae is the most diverse family in terms of NR-PKS production. At the species level (Figure 5c), *Aspergillus nidulans* emerges as the most diversified iT1-NR-PKS producer.

*A. nidulans* FGSC A469 was found to possess six distinct loci (ANIA_08209, ANIA_07825, ANIA_00150, ANIA_06000, ANIA_07071, and ANIA_07909) encoding different iT1-NR-PKSs (Table S5). Corresponding proteins identified via UniProt were Q03149 (wA), Q12397 (StcA), Q5BH30 (MdpG), Q5B0D0 (AptA), Q5AXA9 (PkgA/MdpL), and Q5AUX1 (OrsA). These proteins were analyzed for co-expression using the STRING database across the tree of life, and relevant hits from the subphylum Pezizomycotinaare presented (Figure 5d and Figure S2). Several new species from Sordariomycetes (e.g., *Ophiocordyceps unilateralis*, *Cordyceps javanica*, *Lomentospora prolificans*, *Rosellinia necatrix*), Eurotiomycetes (e.g., *Monascus purpureus*, *Rasamsonia emersonii*, *Exophiala spinifera*, *Cladophialophora carrionii*), and Dothideomycetes (e.g., *Pseudocercospora musae*, *Acidomyces richmondensis*, *Corynespora cassicola*, *Cryomyces minteri*) were predicted to express iT1-NR-PKSs. However, some members of these classes lacked evidence of such expression (e.g., *Stachybotrys chlorohalonata* and *Erysiphe necator* in Sordariomycetes; *Uncinocarpus reesii* in Eurotiomycetes; and *Diplodia seriata* in Dothideomycetes). Interestingly, *Lipomyces starkeyi* from the subphylum Saccharomycotina was also predicted to express at least one (mdpL) iT1-NR-PKS (Figure S2).

## 4. Discussion

### 4.1. Domain Architecture of NR-PKSs Among the Members of Ascomycota

The distribution of the SAT (PF16073), KS (PF00109), AT (PF00698), PT (PF00550), and ACP (PF00975) domains in 17 NR-PKSs are presented in Figure 2. The SAT domain contains the conserved GXSXG motif [24],while the KS domain—bearing the C(H/Q)GS or CHH motif—catalyzes decarboxylative Claisen condensations iteratively (e.g., hexaketide in orsellinic acid vs. octaketide in naphthopyrones) [25,26]. The AT domain selects and loads starter units (typically malonyl-CoA), and unlike reducing PKSs, NR-PKSs typically avoid β-keto reduction [27]. The PT domain, identified as an auxiliary domain in iT1-NR-PKSs (Figure 2), directs first-ring cyclization via a His–Asp catalytic dyad [28]. 

However, the PT domain is used to distinguish NR-PKS subfamilies. For example, orsellinic acid synthase lacks a PT domain, whereas the noranthrone synthase PT domain helps C7–C12 aldol cyclization [27], and the monodictyphenone synthase PT domain helps C6–C11 cyclization [29]. DSL or DXGXXD is highly conserved across all ACP-containing enzymes [26]. The TE domain determines the release mechanism, which may vary across iT1-NR-PKSs, for example, hydrolysis in OrsA [30],Claisen cyclization in WA [25], and dimerization in AptA [31]. Among the 17 iT1-NR-PKSs, only one (Bik1) may contain DH and KR domains (Figure 2c), which may suggest horizontal gene transfer [32] and/or domain shuffling [33].

### 4.2. Phylogenetic Classification of NR-PKSs Among the Members of Ascomycota

Phylogenetic analyses based on KS domain sequences reveal three major clades: clade I (Minimal NR-PKSs): Orsellinic acid synthase [4]; clade II (Standard NR-PKSs with PT and TE domains): Naphtho-γ-pyrone synthase and noranthrone synthase [27]; and clade III (Hybrid/Reductive NR-PKSs): Zearalenone synthase [34]. While Throckmorton et al. (2015) classified NR-PKSs into five phylogenetic clades—clade 1: OrsA; clade 2: PksA; clade 3: MdpG; clade 4: WA; and clade 5: PksP [12]—in contrast, the present study identifies nine distinct NR-PKS clades, six of which are supported by unique KO numbers—clade 1: PksA (K15316), clade 2: Fsr1, clade 3: WA (K15321), clade 4: PksCT, clade 5: Zea1 (K15417), clade 6: OrsA (K15416), clade 7: AptA (K15317), clade 8: MdpG (K15415), and clade 9: Bik1.OrsA appears to represent the ancestral functional archetype, with subsequent diversification driven by modular domain rearrangements and enzymatic partner recruitment [12]. This is further supported by the observation that OrsA is uniquely present in *Aspergillus nidulans* FGSC A4 69 among the 40 species analyzed from the KEGG genome database.

In the phylogenetic tree (Figure 3), Cluster X represents hybrid PKSs, which were excluded from the present analysis. The positioning of hybrid PKSs among NR-PKS clusters in the reference tree suggests that they are chimeric biosynthetic systems, incorporating elements from multiple PKS types. Such hybrids often emerge through horizontal gene transfer via plasmids, transposons, or phages [35,36]. In fungi, hybrid PKSs are known to produce complex metabolites like fumonisins [37] and lovastatin [38].

### 4.3. Incongruency Between Phylogenetic Classification of Ascomycota and Distribution of NR-PKSs

The phylum Ascomycota is one of the most taxonomically and functionally diverse fungal lineages, with a rich evolutionary history reflected in its secondary metabolite biosynthetic capacity. It is divided into three major subphyla based on multigene phylogenies: Pezizomycotina (filamentous fungi and principal producers of NR-PKSs), Saccharomycotina (unicellular yeasts, typically devoid of NR-PKSs), and Taphrinomycotina (basal lineages with limited secondary metabolism) [39]. Within Pezizomycotina, the dominant classes—Sordariomycetes (flask-shaped perithecia), Eurotiomycetes (cleistothecial/gymnothecial ascomata), and Dothideomycetes (pseudothecial ascomata)—exhibit varying degrees of NR-PKS distribution [39]. Contrary to expectations of vertical inheritance, NR-PKS distribution does not align strictly with taxonomic classifications. For example, clade 5: Zea1 (K15417), associated with zearalenone biosynthesis, appears in phylogenetically distant Sordariomycetes and Dothideomycetes but is absent in Eurotiomycetes (Figure 5a). Even within a single class, NR-PKS expression is inconsistent—species such as *Stachybotrys chlorohalonata* and *Erysiphe necator* (Sordariomycetes), *Uncinocarpus reesii* (Eurotiomycetes), and *Diplodia seriata* (Dothideomycetes) show no detectable expression of the six major NR-PKSs in co-expression analyses (Figure 5d and Figure S2). Previous studies have also reported the patchy, non-taxonomic distribution of NR-PKSs. For instance, NR-PKSs are found in some Eurotiomycetes (e.g., *Aspergillus* spp.) but absent in closely related taxa, and conversely, present in distantly related Sordariomycetes (e.g., *Fusarium*) [4]. Gaffoor and Trail (2006) reported Zea1 in *Fusarium* (Sordariomycetes) though absent in other Sordariomycetes [40]. The structural resemblance of Zea1 with *Aspergillus* NR-PKSs suggested horizontal gene transfer (HGT), and subsequent evidence supported transposon-mediated mobilization of PKS clusters between distant taxa such as *Aspergillus* and *Fusarium* [13].

## 5. Conclusions

The present phylogenetic classification delineates fungal NR-PKSs into nine major clades, each supported by distinct gene orthologs and/or associated biosynthetic gene clusters (BGCs). These enzymes represent a functional continuum between classical non-reducing PKSs and hybrid PKS systems, expanding the landscape for novel natural product discovery. Notably, the distribution of NR-PKSs across Ascomycota does not strictly align with established phylogenetic relationships. This incongruence underscores the influence of horizontal gene transfer (HGT), lineage-specific gene loss, and ecological pressures in shaping the evolutionary trajectory of fungal polyketide biosynthesis.

## Figures and Tables

**Table 1:** Identification of the interactive type 1 NR-PKSs within Ascomycota (fungi).

Sl. No.	Cand_Id	Domain Class	Domain Subclass	KO No.	Definition	BGC Product Match
1.	StcA (XP_050468055.1)	Type I iterative cis-AT	non-reducing	K15316	PKSA; noranthrone synthase [EC:2.3.1.221]	Sterigmatocystin
2.	PkgA (Q5AXA9)	Type I iterative cis-AT	non-reducing	K15317	APTA; asperthecin synthase	Alternariol
3.	AptA (Q5B0D0)	Type I iterative cis-AT	non-reducing			Asperthecin
4.	Pks13 (G3KLH6.1)	Type I iterative cis-AT	non-reducing			TAN-1612
5.	VrtA (XP_014544259.1)	Type I iterative cis-AT	non-reducing			Viridicatumtoxin
6.	WA (Q03149) PksP (Q4WZA8)	Type I iterative cis-AT	non-reducing	K15321	WA; naphtho-gamma-pyrone polyketide synthase	Naphthopyrone
7.	MdpG (XP_657754.1)	Type I iterative cis-AT	non-reducing	K15415	MDPG; monodictyphenone synthase	Endocrocin
8.	OrsA (Q5AUX1)	Type I iterative cis-AT	non-reducing	K15416	ORSA; orsellinic acid synthase	Elsinochrome
9.	Pks1 (L7XAV2.1)	Type I iterative cis-AT	non-reducing	K15417	ZEA1; zearalenone synthase	Dehydrocurvularin
10.	HypS (XP_030988075.1)	Type I iterative cis-AT	non-reducing			Hypothemycin
11.	RadS (XP_029743834.1)	Type I iterative cis-AT	non-reducing			Radicicol
12.	Sor2 (G0R6S9.1)	Type I iterative cis-AT	non-reducing	NA	Citrinin synthase	Stipitatic acid
13.	AfoE (A0A5N6H990)	Type I iterative cis-AT	non-reducing			Chaetoviridin
14.	PksCT (Q65Z23)	Type I iterative cis-AT	non-reducing			Citrinin
15.	Bik1 (Q9P855)	Type I iterative cis-AT	non-reducing	NA	Bikaverin synthase	Bikaverin
16.	Fsr1 (XP_044704191.1)	Type I iterative cis-AT	non-reducing	NA	Fusarubin synthase	Fusarubin

Different color codes represent different KO numbers/BGCs.

## Data Availability

All data are available in public databases like the KEGG Orthology database (https://www.genome.jp/kegg/ko.html last accessed on 31 March 2025), UniProt database (https://www.uniprot.org/ last accessed on 31 March 2025), NCBI Genome database (https://www.ncbi.nlm.nih.gov/genome last accessed on 31 March 2025), and NCBI protein database (https://www.ncbi.nlm.nih.gov/protein?cmd=retrieve last accessed on 31 March 2025).

## Supplementary Materials

The following supporting information can be downloaded at: https://www.mdpi.com/article/10.3390/jof11090641/s1, Table S1: List of NR-PKSs reported in Ascomycota; Table S2: Protein sequences of NR-PKSs reported in Ascomycota retrieved from either the UniProt or NCBI Protein databases; Table S3: Identification of the protein domains present in interactive type 1 NR-PKSs; Table S4: Structural validation of the protein 3D models; Table S5: Distribution of iT1- NR-PKSs across Ascomycota obtained from the KEGG genome database; Figure S1: Tertiary structure and Ramchandran analysis: A. StcA (a1. 3D structure, a2. Ramchandran plot), B. Fsr1 (b1. 3D structure, b2. Ramchandran plot),C. WA (c1. 3D structure, c2. Ramchandran plot), D. PksCT (d1. 3D structure, d2. Ramchandran plot), E. Pks1 (e1. 3D structure, e2. Ramchandran plot), F. OrsA (f1. 3D structure, f2. Ramchandran plot), G. Pks13 (g1. 3D structure, g2. Ramchandran plot), H. MdpG (h1. 3D structure, h2. Ramchandran plot), and I. Bik1 (i1. 3D structure, i2. Ramchandran plot); Figure S2: Distribution of different iT1-NR-PKSs across the fungi phylum Ascomycota: phylogenetic tree showing co-expression of wA, stcA, mdpG, aptA, mdpL, and orsA across the class Dothideomycetes of subphylum Pezizomycotina, subphylum Saccharomycotina, and subphylum Taphrinomycotina. [4,41,42,43,44,45,46,47,48,49,50,51,52,53,54,55] are cited in the Supplementary Materials.

## Abbreviations

The following abbreviations are used in this manuscript:

NR-PKSs	Non-reducing polyketide synthases
BGC	Biosynthetic gene cluster
KO	KEGG Orthology
PKS	Polyketide synthase
