# Supplementary material for: Diversity and Distribution of Non-Reducing Polyketide Synthases (NR-PKSs) in Ascomycota (Fungi)
Source: J Fungi (Basel). 2025 Aug 29;11(9):641. doi: 10.3390/jof11090641 (PMC12470490; doi:10.3390/jof11090641)
Supplement: Supplementary file 1 [file jof-11-00641-s001.zip › jof-3724896-supplementary.pdf]

Table S1. List of NRPKS reported in Ascomycetes fungi

Sl. No.	Name	Organism	Product	Role	UniProt ID/NCBI Protein ID	Reference
1.	AfoE	*Aspergillus flavus*	Aflatoxin precursor (norsolorinic acid)	Mycotoxin biosynthesis	A0A5N6H990	[41]
2.	PksP / Alb1	*Aspergillus fumigatus*	1,3,6,8-Tetrahydroxynaphthalene (T4HN) → DHN-melanin	Conidial pigment biosynthesis	Q4WZA8	[42]
3.	AptA	*Aspergillus nidulans*	Asperthecin (red pigment)	Pigment biosynthesis	Q5B0D0	[42]
4.	OrsA	*Aspergillus nidulans*	Orsellinic acid (a precursor to depsides like lecanoric acid)	Pigment biosynthesis	Q5AUX1	[43]
5.	wA (WA PKS)	*Aspergillus nidulans*	YWA1 (precursor to DHN-melanin)	Early-stage melanin biosynthesis	Q03149	[44]
6.	MdpG	*Aspergillus nidulans*	Catalyzes the first step in the monodictyphenone → emodin → austinol pathway	Monodictyphenone biosynthesis	NCBI Protein ID*: XP_657754.1	[45]
7.	PkgA	*Aspergillus nidulans*	Catalyzes the synthesis of alternariol (AOH) and roduces alternariol monomethyl ether (AME) via methylation by downstream tailoring enzymes	Mycotoxin biosynthesis	Q5AXA9.1	[42]
8.	StcA	*Aspergillus nidulans*	Sterigmatocystin biosynthesis	Mycotoxin biosynthesis	NCBI Protein ID*: XP_050468055.1	[45]
9.	Pks13	*Aspergillus niger*	Tensidol B	Biosurfactant biosynthesis	NCBI Protein ID*: G3KLH6.1	[46]
10.	Pks1	*Aspergillus terreus*	Atrochrysone → converted to citreoviridin	Mycotoxin biosynthesis	NCBI Protein ID*: L7XAV2.1	[47]
11.	LovB	*Aspergillus terreus*	Biosynthesis of lovastatin's dihydromonacolin L precursor	Lovastatin	NCBI Protein ID*: AAD39830.1	[48]
12.	BcPKS	*Botrytis cinerea*	Naphthoquinone pigment (botcinone)	Pigment biosynthesis	R9TM73	[49]
13.	Pks1	*Botrytis cinerea*	Pigment Naphthoquinone	Forms coloured polyketide intermediates	Q6RKJ9	[50]
14.	PKS4/ BIK1	*Fusarium fujikuroi*	Bikaverin	A red pigment with antibiotic properties	Q9P855	[51]
15.	Fsr1	*Fusarium poae*	Fusarubin biosynthesis	Fusarubin is a naphthoquinone derived mycotoxin	NCBI Protein ID*: XP_044704191.1	[52]
16.	VrtA	*Metarhizium brunneum*	Viridicatumtoxin biosynthesis	Mycotoxin biosynthesis	NCBI Protein ID*: XP_014544259.1	[53]
17.	PksCT	*Monascus purpureus*	Citrinin (mycotoxin)	Mycotoxin biosynthesis	Q65Z23	[54]

18.	HypS	*Pyricularia grisea*	Resorcylic acid → hypothemycin (via macrolactonization)	A natural antiprotozoal product	NCBI Protein ID*: XP_030988075.1	[55]
19.	RadS	*Pyriculariapennisetigena*	Radicicol biosynthesis	A natural, antifungal macrocyclic antibiotic	NCBI Protein ID*: XP_029743834.1	[55]
20.	Sor2	*Talaromycesmarneffei* (*Penicillium marneffei*)	Mitorubrinol and mitorubrinic acid	Developmental pigment regulation	NCBI Protein ID*: G0R6S9.1	[56]

Table S2. Protein sequences of NRPKS reported in Ascomycetes fungi retrieved from either UniProt or NCBI Protein database

Sl. No	UniProt ID/NCBI Protein ID	Amino Acid Sequence
1.	A0A5N6H990	>tr|A0A5N6H990|A0A5N6H990_ASPFL Polyketide synthase OS=Aspergillus flavus OX=5059 GN=BDV35DRAFT_388808 PE=4 SV=1<br>MANITESLFIFGSQIAYSEKAVSDIRTTLRNKLSRQWVLNTVAELPRYWDALTKELPEIA<br>ESIGSEGRQLLENLGPWLKSNRSKALGVQGDALPGVVFVPLIVVGQLAEYQQYLQACNNG<br>GNSDAQAALVASKTPALGFCMGLLSAYAVASAQNLEELNRYSAVAVRLAMLAGAWSDAQE<br>TWNPHKTYAVGWTSEKQLQDLRRVIESISPEAYISVLFDEARATVTVSERAAARLMRKAR<br>AAGVVLSDTGFRGELHSPKVQAKEKVQDLIAFCNKRHDLQFAKTDHLPLTTFTNAGDGKP<br>LVSGDGSLQGHALETLFARQCDWYGTFKAVKAAYLNGSETSVITFGPDRCLPPTLVRHMG<br>SRAVHFADVYEQPSSADDDLVNDNNPVDDKNPINDNNTINGNTPVNSDKPVNNDEPVNND<br>DPVNDDDAIAIVGMSIKVAGADDLDEFSQLLRKGTSQHEKVTRERLNFDSLFREPDTRDY<br>FCNFVRDVDAFDHKFFKRSPRESAAMDPQHRLLLQAAYQAVEQAGMFTEATRAGQEDKDR<br>NHVGVYIGTPSVDYEHNVATHPLNAFMATGNLQSYLPGRVANYFGWTGPAIAFDTACSSS<br>AVAIHSACNSIRNGECYAALAGGVCILTNPHWFQNLTAASFLSPTGQCKPFDEKGDGYCR<br>AEGIACVFLKRMADARADGNPILGRLASSAVYQNQNHTPIFVPNSPSLAQLFGDVVKKAH<br>LSPRDIALVEAHGTGTAVGDPAEWAALRKAVGGPIRPQPLPVGSVKGHVGHTEGASGIIS<br>LIKVLMMIHEGFIPPQASFNKLNPTIKATAASDMMEIVTSLRSWDSRNKVVLINNYGASG<br>SNAAMVVAAPRTPNERQDAASIKKLEGRFPFCISGSDARAVKEYAAKLATIVNRQSKSTL<br>ADLSFNINRQTNPDLPQRLLLRCRSVDELKDRLASVSEEELTNTQAERPVVLCFGGQVST<br>FVGLDRKLYDNIAVFRHHLDECDNVLQTELGLPSIFPDIFSRTALPDPVRLQVALFAMQY<br>ASARSWIDCGLSGKVVSVIGHSFGELTALCISGVLSLKDALTLVARRAQLVRDAWGDEKG<br>SMMAVEADESLVHSLLEHAGRQLGGLASPASIACYNGPRSFTLAGSVKAIDAVAEAISNG<br>DKFSAVRSKRLNVTNAFHSSLVDPLIDRLEDVGKQLEFHEPSIPLERATETAFAGPLTPK<br>FVPNHMRNPVFFSHAIQRLAKKYPSAIFLEAGSSSTITVMARRALAGQVSKSQHFESVSV<br>TNEKGLDGLTDATVSLWEQGLRVAFWAHHTLQTKDYTHILLPPYQFEKARHWMDYKSASD<br>LVAGMTQRAPVSDSKSMPLWEFVGYQNNDTKHPRFRVNIISDKFKKLVSGHIFAHTAPVL<br>SGTVQSDMAVEALFSIHPDWKEQGMIPTMIEANIPTPICVDSSRKVWIDYEALDAEHTLW<br>QLNISSTSAEGTLPRRHLHGRLHMRSPNDPAFISQFARFERLVPYEQCARVLAEQGGPDV<br>DVLQGRQVYKAWSEFIEYSDQYNAVHAVVGKKNECAGRVHRRHAGESYLDIALHDSFKQV<br>SGLFLNCMSDSKPGEIHIGSDVELVMRSPRGILQPGQTQDIWHVYARHSQVSARVYMADA<br>FIFNPANGELVEVILGLQYTRMSIDSMKRLLMNLTVDEWALNLNKSGAAVAKPTPRAPQV<br>AVSSSPKKAQEKQKETKGPSSQPAKSNLTVEVKRVLASVVGAEVDSITLDSETADIGIDS<br>LMGIELLREINSAFHCKVDLPELLAATTVGHIVTLVAGALSEGDAIELTDDDDDMSESSQ<br>DDRSPTPTTASEIDTATSEDELPPIKDVHSEELTKRDAAVREAEAEKYVAKYTAGWTPAQ<br>PAYTSEAIADSNDAVVIVTGATGSLGVHVVAALASHPSVATVVCINRPRPTPVETRQDEA<br>FSSRGITLTPEARSKLRILSTDTSQPQLGLEASEYEWLTQHGSHIVHSAWPLSGSRPISA<br>FAPQFQTMRNLLDLARDMAVHRRVGFQMVSSLSVVGHAKEPLVSEDRVHMDSVLPPGYGE<br>AKWVCERMLDETLHKYPTLFRAMAVRPAQIAGSSRSGVWNSVEQVPFIVKSAQSLGVWPN<br>FRGTVRWIPVDAVAAILVDLLYIGDEETAPDPYPIYHIDDPVGRPWEQISSVLTDALDIP<br>ASQSVPFKEWLGIVAHSSKPEIENPAGRLADFLEQNFERLSCGGLVLDTTKAQEHSATMR<br>ALGPVSDEVARGYIRAWKEMGFLSS
2.	Q4WZA8	>sp|Q4WZA8|ALB1_ASPFU Conidial pigment polyketide synthase alb1 OS=Aspergillus fumigatus (strain ATCC MYA-4609 / CBS 101355 / FGSC A1100 / Af293) OX=330879 GN=alb1 PE=1 SV=1<br>MEDLHRLYLFGDQTISCDEGLRNLLQAKNHTIVASFIERCFHALRQEITRLPPSQRTLFP<br>RFTSIADLLAQHRESGTNPALGSALTCIYQLGCFIDYHGDRGHPYPSSDDGLLGSCTGML<br>SCTAVSSCKNVGELLPLAVEIVRLTIHLGLCVMRVREMVDSTESSSGSWSILVSEINEAD<br>ATSLIGNFVKKRGIPPSSQPYISAVGSKGLTISAPPEILDNFIEEGLPKEYKHFKAPGVS

		GPYHAPHLYNDREIRNILSFCSEDVILRHTPRVPLVSSNTGKLVQVKSMRDLLKVALEEI<br>LLRKICWDKVTESCLSIVQATNDKPWRILPIASNATQGLVTALQRMGNCQIEVDTGVGAP<br>QMDPAAPNATGNASRSKIAIIGMSGRFPEADGIEAFWDLLYKGLDVHKKVPPERWDVDAH<br>VDLTGTKRNTSKVPYGCWINEPGLFDARFFNMSPREALQADPAQRLALLSAYEALEMAGF<br>VPNSSPSTQRDRVGIFMGMTSDDYREINSGQDIDTYFIPGGNRAFTPGRINYYFKFSGPS<br>VSVDTACSSSLAAIHLACNAIWRNDCDTAISGGVNLLTNPDNHAGLDRGHFLSRTGNCNT<br>FDDGADGYCRADGVGTIVLKRLEDAEADNDPILGVINAAYTNHSAEAVSITRPHVGAQAF<br>IFNKLLNDTNTNPHEIGYVEMHGTGTQAGDAVEMQSVLDVFAPDYRRGPANSLYLGSAKS<br>NIGHGESASGVTSLVKVLLMLKQNMIPPHCGIKTKINHNFPTDLAQRNVHIAFKPTPWNR<br>PVSGKRKMFINNFSAAGGNTALLMEDAPLREITGQDPRNVHVVSVTARSQTALKRNINAL<br>IKYINTHAPSSPANERRFLASLAYTTTARRMHHPFRVTAVGSSVKDIREVLRQRADQDVT<br>TPVPATAPKTGFVFTGQGAQYTGMGKQLYEDCATFRSTIHRLDCIAQSQGFPSILPLIDG<br>SMPVEELSPVVTQLGTTCLQMALVDYWKGLGVTPAFVLGHSLGDYAALNSAGVLSTSDTI<br>YLCGRRAQLLTQQCQMGTHAMLAVKAAVSEIQHLLDPDVHAVACINGPTETVISGLSGRI<br>DELAQQCSSQNLKSTKLKVPFAFHSAQVDPILESFEESAQGVIFHEPAVPFVSALNGEVI<br>TESNYSVLGPTYMVKHCREAVNFLGALEATRHAKLMDDATLWVEVGSHPICSGMIKSTFG<br>PQATTVPSLRRDDDPWKILSNSLSTLHLAGVELNWKEFHQDFSSAHEVLELPRYGWDLKN<br>YWIPYTNNFCLTKGGPVTAEVSAPKSTFLTTAAQKIVECREDGNTATLVVENNIAEPELN<br>RVIQGHKVNGVALTPSSLYADIAQTLVDHLITKYKPEYQGLGLDVCDMTVPKPLIAKSGD<br>QFFRVSAVMSWAEQKASVQVWSVNGDGKKMAEHAHCTVKLFNCAERETEWKRNSYLIKRS<br>VSLLQDKAQTGEAHRMQRGMVYKLFAALVDYDENFKAIQEVILDSNEHEATARVKFQAPP<br>GNFHRNPFWIDSFGHLSGFIMNASDATDSKNQVFVNHGWDSMRCLKKFSGDATYQTYVKM<br>QPWKDSIWAGDVYVFEGDDIIAVYGGVKFQALARKILDTVLPPIGGSKTVGAPAPAPARP<br>IGEKKAPPPIKVTGPPKPNPSNARAASPVVARALEILAAEVGLSEAEMTDSLNFADYGVD<br>SLLSLTVTGRYREELNLDLESSVFMDYPTIKDFKAYLAEKGFCDSSSPEPSSEPESKFSF<br>NSDASSEASSGLTTPGITSPVKHEAPKGGQNKVWKSICSIIAEEIGVSVGDIDPSDNLPE<br>MGMDSLLSLTVLGRIRETLGMDLPAEFFLENPTLDAVQAALDLKPKMVPAATPVSEPIRL<br>LETIDNTKPKTSRHPPATSILLQGNPHTATKKLFMFPDGSGSASSYATIPALSPDVCVYG<br>LNCPYMKTPQNLTCSLDELTEPYLAEIRRRQPKGPYSFGGWSAGGICAFDAARQLILEEG<br>EEVERLLLLDSPFPIGLEKLPPRLYKFFNSIGLFGDGKRAPPDWLLPHFLAFIDSLDAYK<br>AVPLPFNDSKWAKKMPKTYLIWAKDGVCGKPGDPRPEPAEDGSEDPREMQWLLNDRTDLG<br>PNKWDTLVGPQNIGGIHVMEDANHFTMTTGQKAKELSQFMATAMSS
3.	Q5B0D0	>sp|Q5B0D0|APTA_EMENI Non-reducing polyketide synthase aptA OS=Emericellanidulans (strain FGSC A4 / ATCC 38163 / CBS 112.46 / NRRL 194 / M139) OX=227321 GN=aptA PE=1 SV=1<br>MKDNTHSTTLIFFGNEFPNDDLKGLFRCLLRLSKDRRFRQLAAFLEESTLVLKKEVAALP<br>QPLRDLVPHFHTVLPLAELGDFRQGPLGAAMESALLTVLELGMFIGHYEAEGRDWNLLEH<br>NTTLAGLSIGLLAAAGVALSTNLAEVAQNGAECVRVSFRLGVYVSEISRKLEAPQADGTL<br>LSWAHVVTGETKSAIQDELSKYNSESGTPELLKVFISAADKTSVSVSGPPSRMKACFSSS<br>HLLRYSKSFALPVYDGLCHASHLYNEDSINTVINSAESVIPVSRPVQLSLHSSNTGQPFP<br>AATAHELFQAIGKELLTGTIYLDNIIDGIIKRIEGFNPSDLQVETFRTSIVFKSVRAALE<br>GEFPDLEIKITDLIPWAFRDYGPRLPRSFAHSKLAVVGMACRMPGGGNDTELFWEILEQG<br>RDVHTTVPADRFDLSTHYDPSGKTDNAATTPYGNFVDKPGLFDAGFFNMSPKEAEQTDPM<br>QRLALVTAYEALEMAGVVPGRTASSNPKRIGTYYGQASDDWRELNASQNIGTYAVTGGVR<br>AFGNGRINYYFKFPGPSFNVDTACSSGLAAVQVACSALWAGEADTVLAGGLNIITDPDNY<br>AGLGCGHFLSKTGQCKVWDETADGYCRADGIGSVVIKRLEDAEADNDNIIAVVLSAATNH<br>SAEAISITHPHAGNQKDNYRQVIDMAAVNPLDVSYIELHGTGTQAGDAVESESVLDVFAP<br>RSPPRRPDQLLQLGAVKSNIGHGEAAAGIASFLKVLLMYQKNMIPAHIGIHTVINPTIPK<br>DLEQRRVRLTQTNTPWPRLPGKKRIAMVNSFGAHGGNTTVLLEDAPERNKDVARENRSTH<br>TVVISAKSKKSLQANIANLALHLEENPDIDLGDLSYTTCARRIHYTLRVGFAVSSIAGLK<br>EALRKAGEKEALAEVRPTPGDVPPVVFAFTGQGAFYQGIARELFESFSYFRDEVLQLDHI<br>VQRLGFQSIVPVIDGSIGENPSATVSQLSIVVIEIALAHLWTLLLGMQPSAVIGHSLGEY<br>AALVVAGVLSTADGIFLAGRRAQLIEKCCTAGSHAMLSVRASVSEISKLLGNAKYEISCQ<br>NTLNDTVIGGTKANLDAARQVLESSSIKCVPVDVPFAFHTEQVDPVLDQLTRVAETVHFK<br>APSIPIISPLLRSVVFDGKTINSSYLIRATREPVHFAGAIEAAQDLGMVNDKTVWVDVGP<br>HPICASFVRSLIPKARVASSCRRNEDNYATMAKNLVALHLAGCTPVWDEYFRANEKAYNL<br>LTLPKYAWNDVNYWIQYIGTWTLDKAHLKYTGTNGPPQVKPSSSALRTSLIHEIIEETIG<br>EETATLKTVSDLQHPEFLEAVHGHRMNNCGVATSSIWTDMSLTVGEYLYNKLAPGSKVHM<br>NVGELEVLHATVANPAKNCTQNLYLDAHLDLRTQKMSLAWFNVDPATGSKAAESYATGSV<br>RFEADAEKWKSEWERLTHLVLGRIETLESMAKDGQASQLSKALSYALFKNVVDYADHYRG<br>MERVVMHDYEAFCDIKLTPERRGMFHTPPHWIDSVSHLAGLIMNGSDASNTRDYFFVTPG<br>YESFRLLAKLDPDVKYQSYVRMFPLPEANMYGGDLYILQDNQIIGMVGHFKFRRVPRLLM<br>DRFFSAEAASKQSVAASASSAPKTATKHAPLPASKPAQAPAEPTPSSLPTVQAQNTSPPQ<br>QVTPSKPAMNGVKTPEEEKPGKADAEGPNGTTSQPEATGVVGQCLQLIANETGQSVNELT<br>PDATFVQLGVDSLMSLVLSEKFRAELGLEVKSSLFLECPTVGDMMDWLEQYC

4.	Q5AUX1	>sp|Q5AUX1|ORSA_EMENI Orsellinic acid synthase OS=Emericellanidulans (strain FGSC A4 / ATCC 38163 / CBS 112.46 / NRRL 194 / M139) OX=227321 GN=orsA PE=1 SV=1<br>MAPNHVLFFPQERVTFDAVHDLNVRSKSRRRLQSLLAAASNVVQHWTASLDGLERADIGS<br>FEDLVELAERQTTQTRGSIVADLVLLTTVQIGQLLVLAEDDPAILSGHAGARAIPMGFGA<br>GLVAAGVAAAATSADGIVNLGLEAVSVAFRLGVELQRRGKDIEDSNGPWAQVISSATTIA<br>DLEQALDRINASLRPINQAYIGEVMTESTVVFGPPSTLDALAKRPELAHATITSPASALA<br>QVPLHGAHLPPISATMIAASSSQQATELWKLAVEEVANKPIDVHQAVTALIHDLHRANIT<br>DIVLTAIGASTETSGIQSLLEKNGLAVELGQLSPTPRPYGNDLDSIPADAIAVVGMSGRF<br>PNSDTLDEFWRLLETATTTHQVIPESRFNVDDFYDPTRAKHNALLARYGCFLKNPGDFDH<br>RLFNISPREAMQMDPVQRMLLMTTYEALEMAGYSPPTPAAPGDSEQAPPRIATYFGQTID<br>DWKSINDQQGIDTHYLPGVNRGFAPGRLSHFFQWAGGFYSIDTGCSSSATALCLARDALT<br>AGKYDAAVVGGGTLLTAPEWFAGLSQGGFLSPTGACKTYSDSADGYCRGEGVGVVILKRL<br>ADAVRSKDNVIAVIAGASRNCNAGAGSITYPGEKAQGALYRRVMRQAAVRPEQVDVVEMH<br>GTGTQAGDRVETHAVQSVFAPSNGNQREKPLIVGALKANIGHSEAAAGIISLMKAILILQ<br>HDKIPAQPNQPIKMNPYLEPLIGKQIQLANGQSWTRNGAEPRYIFVNNFDAAGGNVSMLL<br>QDPPAFALPAPASGPGLRTHHVVVTSGRTATAHEANRKRLHAYLSAHPDTNLADLAYTTT<br>ARRIHNVHREAYVASSTSDLVRQLEKPLADKVESAPPPAVVFTFTGQGAQSLGMGGALYS<br>TSPTFRRLLDSLQSICEVQGLPTKFLNAIRGSGAEGATVTEVDMQVATVALEIALARYWR<br>SLGIRPTVLIGHSLGEYAALCVAGVLSASDALALAFRRATLIFTRCPPSEAAMLAVGLPM<br>RTVQYRIRDSAATTGCEVCCVNGPSSTVVGGPVAAIQALDEYLKSDGKVSTTRLRVQHAF<br>HTRQMDVLLDELEASAAQVPFHAPTLPVASTVLGRIVRPGEQGVFDANYLRRHTREPVAF<br>LDAVRACETEGLIPDRSFAVEIGPHPICISLMATCLQSAKINAWPSLRRGGDDWQSVSST<br>LAAAHSAQLPVAWSEFHKDHLDTVRLISDLPTYAFDLKTFWHSYKTPAAAVSAASATPST<br>TGLSRLASTTLHAVEKLQREEGKILGTFTVDLSDPKLAKAICGHVVDESAICPASIFIDM<br>AYTAAVFLEQENGAGAALNTYELSSLEMHSPLVLREDIEVLPQVWVEAVLDIKSNAVSVH<br>FKGQTSKGAVGYGSATMRLGQPDSAVRRDWSRIQSLVRARVQTLNRSVRPREVHAMDTAL<br>FYKVFSEIVDYSAPYHAVQEAVIAADFHDAAVTLQLTPTADLGTFTSSPFAVDALVHVAG<br>FLLNADVRRPKNEVHIANHIGSLRIVGDLSSPGPYHVYATIREQDQKAGTSLCDVYTTDS<br>QDRLVAVCSDICFKKLERDFFALLTGATRGRSTKPVAAAPAKSMAKRARQLAPSPSPSSS<br>SGSNTPMSRSPTPSSVSDMVDLGTELLQAVAEQTGVSVAEMKSSPGTTFTEFGVDSQMAI<br>SILANFQRTTAVELPAAFFTNFPTPADAEAELGGSALDDLEEDITKPTPSPEQTQARKQG<br>PAPSQHLLSLVAQALGLEASDLTPSTTFDSVGMDSMLSIKITAAFHAKTGIELPAAFFSA<br>NPTVGAAQEALDDDAEEESAPAQTSTNPAKETTIDSSRQHKLDAAVSRASYIHLKALPKG<br>RRIYALESPFLEQPELFDLSIEEMATIFLRTIRRIQPHGPYLIGGWSAGSMYAYEVAHRL<br>TREGETIQALIILDMRAPSLIPTSIVTTDFVDKLGTFEGINRARDLPEDLSVKERAHLMA<br>TCRALSRYDAPAFPSDRQPKQVAVVWALLGLDNRPDAPIASMGRPGLDIGKSMYEMNLDE<br>FERYFNSWFYGRRQQFGTNGWEDLLGDHIAVYTVNGDHFSMMCPPYASEVGDIVIETVTR<br>AVE
5.	Q03149	>sp|Q03149|WA_EMENI Conidial yellow pigment biosynthesis polyketide synthase OS=Emericellanidulans (strain FGSC A4 / ATCC 38163 / CBS 112.46 / NRRL 194 / M139) OX=227321 GN=wA PE=1 SV=2<br>MEDPYRVYLFGDQTGDFEVGLRRLLQAKNHSLLSSFLQRSYHAVRQEISHLPPSERSTFP<br>RFTSIGDLLARHCESPGNPAIESVLTCIYQLGCFINYYGDLGHTFPSHSQSQLVGLCTGL<br>LSCAAVSCASNIGELLKPAVEVVVVALRLGLCVYRVRKLFGQDQAAPLSWSALVSGLSES<br>EGTSLIDKFTRRNVIPPSSRPYISAVCANTLTISGPPVVLNQFLDTFISGKNKAVMVPIH<br>GPFHASHLYEKRDVEWILKSCNVETIRNHKPRIPVLSSNTGELIVVENMEGFLKIALEEI<br>LLRQMSWDKVTDSCISILKSVGDNKPKKLLPISSTATQSLFNSLKKSNLVNIEVDGGISD<br>FAAETQLVNQTGRAELSKIAIIGMSGRFPEADSPQDFWNLLYKGLDVHRKVPEDRWDADA<br>HVDLTGTATNTSKVPYGCWIREPGLFDPRFFNMSPREALQADPAQRLALLTAYEALEGAG<br>FVPDSTPSTQRDRVGIFYGMTSDDYREVNSGQDIDTYFIPGGNRAFTPGRINYYFKFSGP<br>SVSVDTACSSSLAAIHLACNSIWRNDCDTAITGGVNILTNPDNHAGLDRGHFLSRTGNCN<br>TFDDGADGYCRADGVGTVVLKRLEDALADNDPILGVINGAYTNHSAEAVSITRPHVGAQA<br>FIFKKLLNEANVDPKNISYIEMHGTGTQAGDAVEMQSVLDVFAPDHRRGPGQSLHLGSAK<br>SNIGHGESASGVTSLVKVLLMMKENMIPPHCGIKTKINHNFPTDLAQRNVHIALQPTAWN<br>RPSFGKRQIFLNNFSAAGGNTALLLEDGPVSDPEGEDKRRTHVITLSARSQTALQNNIDA<br>LCQYISEQEKTFGVKDSNALPSLAYTTTARRIHHPFRVTAIGSSFQEMRDSLIASSRKEF<br>VAVPAKTPGIGFLFTGQGAQYAAMGKQLYEDCSHFRSAIEHLDCISQGQDLPSILPLVDG<br>SLPLSELSPVVVQLGTTCVQMALSSFWASLGITPSFVLGHSLGDFAAMNAAGVLSTSDTI<br>YACGRRAQLLTERCQPGTHAMLAIKAPLVEVKQLLNEKVHDMACINSPSETVISGPKSSI<br>DELSRACSEKGLKSTILTVPYAFHSAQVEPILEDLEKALQGITFNKPSVPFVSALLGEVI<br>TEAGSNILNAEYLVRHCRETVNFLSAFEAVRNAKLGGDQTLWLEVGPHTVCSGMVKATLG<br>PQTTTMASLRRDEDTWKVLSNSLSSLYLAGVDINWKQYHQDFSSSHRVLPLPTYKWDLKN<br>YWIPYRNNFCLTKGSSMSAASASLQPTFLTTSAQRVVESRDDGLTATVVVHNDIADPDLN<br>RVIQGHKVNGAALCPSSLYADSAQTLAEYLIEKYKPELKGSGLDVCNVTVPKPLIAKTGK<br>EQFRISATANWVDKHVSVQVFSVTAEGKKLIDHAHCEVKLFDCMAADLEWKRGSYLVKRS

		IELLENSAVKGDAHRLRRGMVYKLFSALVDYDENYQSIREVILDSEHHEATALVKFQAPQ<br>ANFHRNPYWIDSFGHLSGFIMNASDGTDSKSQVFVNHGWDSMRCLKKFSADVTYRTYVRM<br>QPWRDSIWAGNVYIFEGDDIIAVFGGVKFQALSRKILDIALPPAGLSKAQTSPIQSSAPQ<br>KPIETAKPTSRPAPPVTMKSFVKKSAGPSVVVRALNILASEVGLSESDMSDDLVFADYGV<br>DSLLSLTVTGKYREELNLDMDSSVFIEHPTVGDFKRFVTQLSPSVASDSSSTDRESEYSF<br>NGDSCSGLSSPASPGTVSPPNEKVIQIHENGTMKEIRAIIADEIGVSADEIKSDENLNEL<br>GMDSLLSLTVLGKIRESLDMDLPGEFFIENQTLDQIETALDLKPKAVPTAVPQSQPITLP<br>QSQSTKQLSTRPTSSSDNHPPATSILLQGNPRTASKTLFLFPDGSGSATSYATIPGVSPN<br>VAVYGLNCPYMKAPEKLTCSLDSLTTPYLAEIRRRQPTGPYNLGGWSAGGICAYDAARKL<br>VLQQGEIVETLLLLDTPFPIGLEKLPPRLYSFFNSIGLFGEGKAAPPAWLLPHFLAFIDS<br>LDAYKAVPLPFNEQEWKGKLPKTYLVWAKDGVCPKPGDPWPEPAEDGSKDPREMVWLLSN<br>RTDLGPNGWDTLVGKENIGGITVIHDANHFTMTKGEKAKELATFMKNALGVCERRLV
6.	NCBI Protein ID*: XP_657754.1	>XP_657754.1 protein mdpG [Aspergillus nidulans FGSC A4]<br>MPVYTPQSGSLPEYSKMKLLYFSNELPKDDLQGLFRRLYNHSKDRRYPLLARFIHEATLAVREEVRQLPT<br>AVKALVPAFETVLNLADYPELRKGPLGGSLEGVLLCVLEIATLIGHVPRLYFKEAADCCSYYENASERFD<br>LHAVSTYLAGLGLGLLSTAAVALCSALADVPVIGAEVVRVSFRLGTLVDEISQNLEPRDTSGSPDTWASV<br>VPGAKVEEVQAELDAIHAREKTPQPSKIFISAWNEGSVTISGPPSRIRRVLRLSEFFRRHRVVSLPVYSG<br>LCHAKHLYNEQHAREIISTRSMDSINALYSPAIPVYQTSTGRPFTASTAKGLFEQLVLELLTQPILWDNV<br>VQGVVDQAYATSATSCDVLVFRISVPINDLRTALGSKLQGFETSTEELIPWILQKSDMEIPRGTAQSKIA<br>IIGMSCRMPGGATDTEKFWELLEQGLDVARKIPADRFDVETHYDPKGKRVNTSHTPYGCFIDEPGLFDAP<br>FFNMSPREAQQTDPMQRLAIVTAYEALERAGYVANRTPATNLHRIGTFYGQASDDYREVNTAQEISTYFI<br>PGGCRAFGPGRINYFFKFSGPSFSCDTACSSSLATIQAACTSLWNGDTDMVVAGGMNVLTNSDAFAGLSH<br>GHFLSKTPGACKTWDVNADGYCRADGIGSIVMKRLEDAEADNDNIIGIIRAAATNHSAEAISITHPHAGA<br>QAYLYRQVMSSAGIDPLDVSFVEMHGTGTQAGDSVEITSITDIFAPITKRRSAQQPLHIGAVKANVGHGE<br>AVAGVTALLKVLLMYQKNAIPPHVGIKNSLNPLFPKDLDKRNLHIPYQKVPWPRVKGKKRYAVVNNFSAA<br>GGNTTVCLEEPPLRETDYVDPRTAHVVNVSAKSKISFKKNLERLVAYLDANPDTSLASLSYTTTARRYHH<br>NHRASVAATDIAQVKKKLLSYIDKVEAHKPIPATGPPQVAFAFTGQGASYKSMNLELFHHSPYFRSQLLH<br>LDALAQGQGFPSFIPAVDGSHEKDYAHSPVVTQLALVSVEIALAKYWISLGVKPNAVVGHSLGEYAAFHV<br>AGVLSASDALFLVGRRAQLLEEKCQIGSHKMLAVRAPLADIEKALEGTNYEVACINGPKETVLSGSQAQV<br>EVVSEILQSVGYRCTSLDVAFAFHSSQTEAILDDFEEAAKDGVLFRAPNMPVISPLLGKVIFDDKTITAK<br>YMRRATRETVNFLSALEMAQTFSTVDEETVWVEIGPHPVCMGFVNATLPAVNETVASMKRGEDNWVTLCN<br>SLTALHCAGVPIEWNEYQRPFEKGLRLLDLPTYAWNDKTYWIQYNGDWALTKGNTFYDAEKSLKAQQTGQ<br>LASVPSGLRTSTVQQIIEESFNGSAGKVVMQSDMMQPDFLDAAHGHKMNGCGVVTSSIHGDIGFTLGGYL<br>YKNLVKGGKAPDMNMANLVVLRGLVAQKNTKKPQYIRVTISTTDINSGVAELIWQNVLNDNTADEPFASA<br>SILYDDAALWLKSWIPSTHLVQGRIEALERLAEDGIANRFTRNMAYLLFANNLVDYAQKYRGMQSVVLHE<br>LEAFADITLSTEKSGTWTIPPYFIDSVAHLAGFVMNVSDAIDTKANYCVTPGWKSLRFAKPLVAGAKYRS<br>YVKMIQTEEDPTVYLGDVYIMQDGAIIGMCGGIQFRRYPRILLNRFFTAPEEAGAISHAAASSTPAPRTK<br>PEPVPVATPATAAAPVAQSPAAPASVTPAPAPAPAPGPTPAAAPAAAGESDSVAAKALVLIAKEAALELS<br>DLTDDASFANLGVDSLMSLVIAEKFREELGVTVTGSLFLEYPTIGDLRSWLLEYYN
7.	Q5AXA9.1	>sp|Q5AXA9|PKGA_EMENI Non-reducing polyketide synthase pkgA OS=Emericellanidulans (strain FGSC A4 / ATCC 38163 / CBS 112.46 / NRRL 194 / M139) OX=227321 GN=pkgA PE=1 SV=1<br>MTPSASPSPSPNPGDANVKVDADSPNDFILFSHELPSGDIQDLIRRLHRYGTLPGYPHLA<br>RFLQECALLLRTEIQKLPRALRDSVPPFHDVVTLASHWDRLKSGPLSGAWDGPFLCLYEI<br>AMLIGHHETHQLSYRRPACLVGISVGLFSAAAVAVSKSISDLVSYGAESVRTAFAFCVHV<br>QRVSQELEPTMTEQAASVSWATVVIGVPADTIQVELDRFNHLKESESPGANARPLTGVSI<br>SHVDQTSVGVTGPPSRLKQLFRQSELLRSSRHSALPISGGLCHVPNVYDDEDVRAILEMA<br>EVWEKWGTRALQVPLISPFTGSPFLCPDAYHLIEAICTEALTKQLYFDKLAGGVVTQLNG<br>LSCQVLHYGASLMSDTIIDDVTSQLSPCDTARQCLVDWALRDAFDQLPGGPTPPRDAKLA<br>VVGMACRMPGGADTPDHFWELLMNGVDTHTTVPPDRFDLDAHFDPSGEKENTTTKGSQSN<br>RPLSRQAEQTDPMQRLALVTAYEALEMAGFVPNRTPSSHLSRVGTYYGQASDDYREVNAG<br>QKIGTYGIPGTERGFGNGRINYFFNFQGPSFNIDTACSSGLAAVQAACSALWAGEADTVV<br>AGGLNVITSPDIYCMLSKGHFLSKTGQCKVWDIGADGYCRADGIGSVVIKRLDDALADND<br>VILACISAGATNHSAESISITQPHAAAQRENYRQVMDRAGVSPLDVSFVELHGTGTQVGD<br>AVESESVLSFFAPLGRRSHPDKRLHLGAVKSNIGHGEAAAGIASLIKVLLMYRNNTIPRH<br>IGIRTAMNPVVAQHLANRNAGILSENHPWLAATASKKRYAIVNSFGAHGGNTTLLLEDAP<br>SQHSQRYKNHSRRVVASSEVVCTSAKSKASLRANIRALLAYLDTHQETDLRDLAYTTSAR<br>RMHHHIRIASSVTSTAQLRSFLQAAADDVDAYAKHIATATKRTAVFAFSGQGCLYHGAAA<br>HLFEQAPLFRNQVLQLDRIVRRLGFPSILVTVAGDAASVYDSARCPHRESTPSSDASHDS<br>NTNRTSTAPAVDSPLIAQLALVVIQIALVQYWGLLGIKPSVVIGHSLGEYAALVAASVLS<br>VADALFLVGKRAELMLAVCEPGSHAMLSVRGASVDRIEELCRESEKRYPFEVSCVNGLTD<br>LVVTGLRGDMASLRDLLQGSGLKCVLLDIPFAFHSKQMSPILEDFENAAQQITFQEPAVP<br>VISPLLGKCISEANVINGKYLARATREPVDFVAALDSAWADGTVNDKSIWIDIGPHPVCT<br>SFASNHYGKAATQSFASLRRGDETLSTLTATLAALHCLGLPVDWNEYYDLRENPARLLHL<br>DSYQWNYKNYWIPYEGSWTLDKAHAGQNNKTKDDNSAVTPAFFTSSVQQIIFEEYDESMG

		RMEALSDLHHPDLQGAADGHKIHGRSVVTGSIWADITLTVGEYLYKQMVPGGKMPHMNVK<br>GMEVLEAQVLHPDMSQFIQIEGVLDLPRQQTAVRLYAASANGTRNTDKPFASATVCYEEA<br>QDWQDQWQMTSHLVAARANSFWEMAAGGSDDNARPAGKGGPRVNNFFRSIAYQLFANVVD<br>YGARYRGMQRVALSEDTLEATADIVLDKDRHGTWHTPPHWIDSAFQLAGFVMNSFGVQGD<br>GKISGSSRDFFYITPGWRHFRLLERLEPGPEATYRSFVRMFPVGSEPGAYSGDIYLHRGK<br>RLVGVCAGIKFKAVPRALMPVLFPRIEPQAGKRRNQAQTAENRLVKGKGNCEYTHTVFQE<br>PKSRTPAYDVTKSQPHAEHCDISISQKTQPVAAVPVTQPALPAKERGEQNQGQSQSQNAQ<br>ATACLSLISDETGLDLDDLTGEAAFADLGVDSLMSLALSAKIRAELGIDVQSSIFLECPT<br>VQDLVTWLSK
8.	NCBI Protein ID*: XP_050468055.1	>XP_050468055.1 sterigmatocystin biosynthesis polyketide synthase stcA [Aspergillus nidulans FGSC A4]<br>MASHAEPTRLFLFGDQTYDFVADLRDLLNIRNNPILVAFLEQSHHVIRAQMIRELPPKEHKQARTASLAE<br>LLQKYVDRKLPSAFQTALSCVTQIGLFMRQFDDPRVLYPHANDSYVLGVCTGSLAAAAISCSTSLSELLP<br>IAVQTVLVAFRLGLWAEKVRDNLEISETNQTQPWSAVCHVPPEEVAIAIDRFSHKKVRSPVYRAQRPWIT<br>ATSAKTTTVSASPDILSQLASQAPFTNSKLWREIPIYVPAHNNHLFSSRDVDDILATTNENPWSTFGAQI<br>PFLSSVTGKLAWVRNYRDLLHLALSQCLIEPIRWDVVEAEVPRLLKDRDGLDTLTIVAFTTVLSKSLSNA<br>LVTEGIKPAEPPTSINKTPERYSHRPGSDRGKLAIVSMSGRFPEAPSTDSFWDLLYKGLDVCKEVPLRRW<br>DVKTHVDPSGKARNKGATRWGCWLDFAGEFDPRFFSISPKEAPQMDPAQRMALMSTYEAMERGGIVPDTT<br>PSTQRNRIGVFHGVTSNDWMETNTAQNIDTYFITGGNRGFIPGRINFCFEFSGPSYSNDTACSSSLAAIH<br>LACNSLWRGDCDTAVAGGTNMIFTPDGHTGLDKGFFLSRTGNCKAFDDAADGYCRAEGVGTVFIKRLEDA<br>LAENDPILATILDIKTNHSAMSDSMTRPFKPAQIDNMSALLSTAGISPLDLSYIEMHGTGTQVGDAVEME<br>SVLSLFAPDETFRPRDKPLYVGSAKANIGHGEGVSGVTSLIKVLLMMKNDTIPPHCGIKPGSRINRNYPD<br>LPARNVHIAFEPKPWPRTDTPRRVLINNFSAAGGNTAVLVEDAPVRDPVTASDPRTSHIVTVSGHVGKSL<br>KLNLEKLRDHLVKRPEINPSELSYTTTARRWHHPHRVSITGANTMEILRNVESAIARGHGVNRPATKPKI<br>VIACSGQGSQYTGMGWQLYNSYPTFRSDLERFDQLARSYGFPSFLEVYTSKPVGDSMEDLLPVIVQLALV<br>SLEMALGNLLGSFGLKPSAVIGHSLGEYAALYISGVLSAADTLYLVGMRAKLLQERCQRGTHAMLAVRAS<br>PVTLCEVLAESNCEVACHNGPNDTVLSGPLKEVMNLQNSLSATGIKGTLLKLPFAFHSAQVQPILEEFKN<br>VARGVTFHKPQIPVLSPLLVKVIDEKGTVDPVYLARHCREPVKMVSVLEHARDQHIITDRTIVIDVGPKA<br>LMAGMIKTTLDKDTSSALPTLGPSLDVWKSLTNILGTLYSRGLDINWVAYHEPFGSAKKVIELPSYGWDL<br>KDYFIPYKGEWCLHRHEIRCSCATPGKETATSDYQLPSDEQVAAKRPSKQDESKEAYPEIVATTTVHRVV<br>EEKTEPLGATLVVETDISRPDVNQIAQGHLVDGIPLCTPSVYADIALHVGRYSMNRLRASHPGAMDGVVD<br>VADMVIDKALIPHGKSPQLLRTTLTMTWPPKAAATTRSAKIKFATYFADGKLDTEHATCTVRFTSEAQLK<br>SLQKKVPEYQERIKKLGEGLRQGQFIRYTTKSGYKLMSSMASFHRDYKLLNHLILNEADNEAVSTMDFSA<br>AKSEGTFAAHPAYVDAITQVGGFAMNANDNTDIQQEVFVNHGWDSFQVYQPLVKGKTYEVYVRMTEDEKG<br>DLVHGDTIVLYGDAVVAFFKGLSLRRVPRRGLRMVLQQASDKAARLHGNQQAVKTQAPQRAALKQKPQSS<br>PTQPHASKVAYSRSATSPTAGKPVVAARDLSREGDDKFKAVLSVISEESGVALGELTADTNFADIGIDSL<br>SSMVIGSRLREDLGLELGAEFSLFIDCPTVRSLKTLLSGSAVSVNNDKDELEPGQEAETAAPEQLDLRIG<br>DAAPSKVRDANIEPLDLGDELFRNVLRIVSEESGVALDELSAETVFADIGIDSLSSMVITSRFREDLGMS<br>LDSSFNLFEEVPTVARLQEFFGTTSGSTTGSSGSGSSEDETDSIPSTPEEYTTADTRVPECRPTTSVVLQ<br>GLPQMAKQILFMLPDGGGSASSYLTIPRLHADVAIVGLNCPYARDPENMNCTHQSMIQSFCNEIKRRQPE<br>GPYHLGGWSSGGAFAYVTAEALINAGNEVHSLIIIDAPVPQVMEKLPTSFYEYCNNLGLFSNQPGGTTDG<br>TAQPPPYLIPHFQATVDVMLDYRVAPLKTNRMPKVGIIWASETVMDEDNAPKMKGMHFMVQKRKDFGPDG<br>WDVVCPGAVFDIVRAEGANHFTLMTKEHVVYLVRELIDRVMG
9.	NCBI Protein ID*: G3KLH6.1	>sp|G3KLH6.1|ADAA_ASPNG RecName: Full=Non-reducing polyketide synthase adaA; Short=NRPKS adaA; AltName: Full=2-acetyl-2-decarboxamidoanthrotainin biosynthesis cluster protein A<br>MSAPTKLVFFGNEFPNDDLKALFRGLHRHGKDRRFRQLATFLEESTRVLQNEVAQLPEPLKKLVPHFENL<br>MPLTEVDFRQGPLGAAMESALLTILELGMFIGHYEAEERVWDLSADRATLAGLSIGLLAAAGVALSTHLA<br>EVVQNGAECVRVSFRLGVYVHDISRKLEAPQADGSLLSWAHVVTGETASDLQEELSRYNTETGTPELLKV<br>FISAADKTSVSVSGPPSRIRAAFRASQRLRYSKSLALPVYDGLCHAAHLYDEETIHRVLHPDGSVIPTSR<br>PVQLALLSSRSGQPFEATTAAELFRAISTELLTGTIFLDNITAGILDRTERCADATQCQIETYRTSLVFK<br>GLLKALEACFPDRTISTTDLIPWVFQDYGARQPKSCADSKLAIVGMACRMPGGANDLDLFWELLAQGRDT<br>HTTVPADRFDLETHYDPTGETENATRTPFGNFIDQPGLFDAGFFNMSPREAEQTDPMHRLALVTAYEALE<br>MAGIVSGRTPSSNPKRIATFYGQASDDWRELNASQNIGTYAVPGGERAFANGRINYFFKFGGPSFNLDTA<br>CSSGLAAVQAACSALWAGEADTVLAGGLNIITDPDNYAGLGNGHFLSRTGQCKVWDQSADGYCRADGVGS<br>VVIKRLEDAEADNDNILAVVLSAATNHSAEAISITHPHAGAQKENYTQVLHQAAVNPLDISYVELHGTGT<br>QAGDAQEAESVLDIFAPRNHRRRADQPLHLGAVKSNIGHGEAAAGIASLLKVLLMYQKNEIPAHIGIPTV<br>INPAIPTDLEQRKVYLPRTKTAWPRAAGQIRRAIVNSFGAHGGNTTLVLEDAPEKQVTVAREERSTHPVV<br>ISAKSKKSLAANVETLLAYLDENPETDLGDLSYTTCARRMHHSWRLATAVSDIPALQKFLRNAVSNDAVS<br>QTRPIPTEAPPVVFTFTGQGAYYAGLAQGLFQALPFFRAEVRQLDHLSQRLGFPSIVPVILGEVEEGTAT<br>ALVTQLSIVIVEIALARLWLLLLGIPAPHAVIGHSLGEYAALAVAGVLSTADALYLVGHRAQLIEEHCTP<br>GSHAMLSVRATIADIERLVGTGSDAPTYELSCQNTHQDTVIGGSIQDLNAIREKLEHEGIKCVNVDVPFA<br>FHTAQMDAVRERLAKAVAAVPFKTPSVPVLSPLLGSVVFDGKSINPEYIVRATREPVQFATAIDAAQELG<br>IVNSQTLWVDIGPHPICASFVRSLVPGARIVSSCRRNEDNFATMAKSLCTLHLAGRTPSWAEYFRPDEQA<br>YSLLRLPKYRWNEVNYWIQYLGTWTLDKAHLKNGGSQKRAITDVPSISSLRTSLIHQVTEETVDKTTATL

		KAISDIQHPDFLEAVHGHTMNNCGVATSSIWTDMAMTVGEHLYRRLVPGTDHVLMDLCDFEVQHAQVANT<br>NSNTPQPLALEAHLDLPTRHMSLAWYDVNATTNQRADAPFATGSIKYPADPTGAAWSIEWSRITHLIQGR<br>IEALQHLAAENKASTLSKPLAYALFKNVVDYAPRYRGMDRVVIHDHEAFSDITLTTDRHGTWHTPPHWID<br>SVSHLAGLVMNGSDASNTRDFFYVTPGCSSCRMAEPLIAGGKYRNYVRMFPMPDEAHMYAGDLYILREDK<br>IIGVVEQLKFRRVPRLLMDRFFSPNKNAAAHAAPAPAPAAVPAVKKQPPTETIQPQAPKTEQKQDQLQLP<br>NLASAAPSTASSSSSPSSSGVATPTTEQEAPGADASAVTGVAGKCLELIANETGLGVAELTADATFVQLG<br>VDSLMSLVLSEKLRSEMGLEIKSSLFLECPTVGDLTGWLEQYC
10.	NCBI Protein ID*: L7XAV2.1	>sp|L7XAV2.1|CURS2_ASPTE RecName: Full=Non-reducing polyketide synthase curS2; AltName: Full=Dehydrocurvularin biosynthesis protein 2<br>MDSNRPAVLLFGDVTDPWVDGIDYVYSQAATTPWLRSFLRDLFTVLKVEMRTMDRTLQESFRDCGSFQEL<br>AERYRHTGDDFGMAHAMLTYVIRAVVLLETISAEPHLLDSNRPRPELIGVSGGLFSAAVVSVSTNFQSLY<br>DTCLEAGRVWARLCNLTLVKSRAIEERPGTWGWAVLGIPAGKLSQTLEQFQNDMGVPSAKRARVGVTGDR<br>WSTVIGPPSILELVLNECPTLKNLPKNELDIHALQHTLDISNADIDYIVGDSSLLDTPLPQGYRIYGLDD<br>DRPEATYPSWSHLLRASASQTLARPLNIVQAVSKLNAALGTSTHVDFKIMGPSSHAAYIAKVLQTAGREV<br>SLQDRITAKPPTPDSRDGIAIVGMAGKGPGSDDLEEFWDVLLKGLDLHQEVPPDRYDLDEYYSPKHPPAG<br>PGKCTMTCRHGCFMNNPGHFDSKFFHISPREALLMDPAHRLFLMNAYEALEMAGYSDGQTKMTDPTKIAT<br>FFGQCNDDWHVVGHRTLGCDAYTLQAVQRAFGPGRLAFQFNWEGPTYALDSACAATSSCIHLACMSLIAR<br>DIDMAVAGAANVLSTPHSFTSLSRSGVLSDSGNCKTYRDDADGYCRADFSGAVVLKRLDDAIAHNDNILA<br>VISSSARNHSGNSTSITTSDAAAQERLFHKVLRNARVTPEDISYVEMHGTGTQVGDKAEMGAVSSVFSKR<br>RDGELLPVGAIKANLGHSEAAAGMSSLLKSILMFQKGTIPPQAGMPHTLNPNFPPLHEINIKIPEEPLEF<br>KSVGGKPRRILLNNFDAAGGNACLLLEDYTHTKEREADVRSAHTIVTSARTQASHLLNKQRLLKWLRSNP<br>NTRIEDLAYTTTARRMHHPIRFALTASTTQEAISKLESEIERNTNSPASRHVPVVFVFTGQGSHYAGMGA<br>ELYRTSSAFRERVDLCVDICAGNNFAPFVDIITDEGVDVSSKTAAQVQLAVLTLEMALTHFWRLAGIEPA<br>MVMGHSLGEYAALHAAGVLSLADALYLVGHRALILQERCESGSCSMLSVSTSVANVREQLSQLQSSSCGV<br>ACINSPSSTVVSGIAEDLAEFQANITAQDAKVRTKTLSIPFAFHSFQMDPILQDYGTIAAGVTFSAPKIP<br>VASTLLGSIVGEPGVFDHDYLVQQTRQPVNFVGGLNAVQSKLSDPLWLEIGPSPVCVSFVRDTLSPSLSK<br>LTHTLQPNTHNWASISKSLAAAYINGVDIDWVALHAPYETNLQLLTLPSYAWDVKDYWITHTDRVTEAVP<br>EQSPVTGSGPLVSTCAQYLVSKSSSPKVQVVFRASISDPGFMELIDGHKMQGIGLCSGSVFCEAAFAAAK<br>YALEYSGRRNVTQPWLTLQKPELLLPLTKKLAGADGSLITTAVMDSPSAHRISVTFKLTSGSESHELGSC<br>IVNFRDPAKTQADWDRVSYFIQARMDEVIKNAKEGPGHRMQPEVFYALFANAVEFSTDFQGVDEAYIAKD<br>FQEAAALVTLPHDPAGTKFTFSPYWGEALVHLAGFMVNGNPSKSPQKTFIVMGFESVEQTVPLVPGKKYM<br>AYTRISKWVKETAYCDAVVFDPETSNIILQCIDLRYQELPRVTWKHVLDGPHGGSSAHGHKAPVQETKKA<br>VEVSRQAVAVAPVQPQPAEVGEDDDDDFDEGLVDAILDSISKATGSDPSEFTDDTIVADLGVDSIMAIEV<br>VATVKEQSGLDLPATFVLEHPTIGDLRRAFGANKPKTSKPQPGSTTPSSSQSSIPSSPNPEPTSMSDTAS<br>SLGSSLVDIEKDQTFTPPPELEPKPNHHLGKMDETDTSPAPTVRITLLQGRPSPKRTPFYMMADGTGTIA<br>TYIHLPAFKSKMPVYGIDSPFLRCPSRLTKDVGIPGVAKLIVDALVTAQPTGPLMIGGFSAGSIVAYEVT<br>RQLGALGRQVTGLVLIDMCCPRSSLLDEDAMNSEDDASFAIFENAVSKDGLWSLASTTQDHFRAYHVAMH<br>AYHPPYMTAQERPSHTAVIWAEKGMVNRVVGNDRLMQMLADQGIPTTSYPGYMEDPRLGAFACLVPDRTA<br>ADLGPNGWEKYTAGEVLALSVAGDHLDLPMPGHVHLLQREMEKAFAYFEGEST
11.	NCBI Protein ID*: AAD39830.1	>AAD39830.1 lovastatin nonaketide synthase [Aspergillus terreus]<br>MAQSMYPNEPIVVVGSGCRFPGDANTPSKLWELLQHPRDVQSRIPKERFDVDTFYHPDGKHHGRTNAPYA<br>YVLQDDLGAFDAAFFNIQAGEAESMDPQHRLLLETVYEAVTNAGMRIQDLQGTSTAVYVGVMTHDYETVS<br>TRDLESIPTYSATGVAVSVASNRISYFFDWHGPSMTIDTACSSSLVAVHLAVQQLRTGQSSMAIAAGANL<br>ILGPMTFVLESKLSMLSPSGRSRMWDAGADGYARGEAVCSVVLKTLSQALRDGDTIECVIRETGVNQDGR<br>TTGITMPNHSAQEALIKATYAQAGLDITKAEDRCQFFEAHGTGTPAGDPQEAEAIATAFFGHEQVARSDG<br>NERAPLFVGSAKTVVGHTEGTAGLAGLMKASFAVRHGVIPPNLLFDKISPRVAPFYKNLRIPTEATQWPA<br>LPPGQPRRASVNSFGFGGTNAHAIIEEYMEPEQNQLRVSNNEDCPPMTGVLSLPLVLSAKSQRSLKIMME<br>EMLQFLQSHPEIHLHDLTWSLLRKRSVLPFRRAIVGHSHETIRRALEDAIEDGIVSSDFTTEVRGQPSVL<br>GIFTGQGAQWPGMLKNLIEASPYVRNIVRELDDSLQSLPEKYRPSWTLLDQFMLEGEASNVQYATFSQPL<br>CCAVQIVLVRLLEAARIRFTAVVGHSSGEIACAFAAGLISASLAIRIAYLRGVVSAGGARGTPGAMLAAG<br>MSFEEAQEICELDAFEGRICVAASNSPDSVTFSGDANAIDHLKGMLEDESTFARLLKVDTAYHSHHMLPC<br>ADPYMQALEECGCAVADAGSPAGSVPWYSSVDAENRQMAARDVTAKYWKDNLVSPVLFSHAVQRAVVTHK<br>ALDIGIEVGCHPALKSPCVATIKDVLSGVDLAYTGCLERGKNDLDSFSRALAYLWERFGASSFDADEFMR<br>AVAPDRPCMSVSKLLPAYPWDRSRRYWVESRATRHHLRGPKPHLLLGKLSEYSTPLSFQWLNFVRPRDIE<br>WLDGHALQGQTVFPAAGYIVMAMEAALMIAGTHAKQVKLLEILDMSIDKAVIFDDEDSLVELNLTADVSR<br>NAGEAGSMTISFKIDSCLSKEGNLSLSAKGQLALTIEDVNPRTTSASDQHHLPPPEEEHPHMNRVNINAF<br>YHELGLMGYNYSKDFRRLHNMQRADLRASGTLDFIPLMDEGNGCPLLLHPASLDVAFQTVIGAYSSPGDR<br>RLRCLYVPTHVDRITLVPSLCLATAESGCEKVAFNTINTYDKGDYLSGDIVVFDAEQTTLFQVENITFKP<br>FSPPDASTDHAMFARWSWGPLTPDSLLDNPEYWATAQDKEAIPIIERIVYFYIRSFLSQLTLEERQQAAF<br>HLQKQIEWLEQVLASAKEGRHLWYDPGWENDTEAQIEHLCTANSYHPHVRLVQRVGQHLLPTVRSNGNPF<br>DLLDHDGLLTEFYTNTLSFGPALHYARELVAQIAHRYQSMDILEIGAGTGGATKYVLATPQLGFNSYTYT<br>DISTGFFEQAREQFAPFEDRMVFEPLDIRRSPAEQGFEPHAYDLIIASNVLHATPDLEKTMAHARSLLKP<br>GGQMVILEITHKEHTRLGFIFGLFADWWAGVDDGRCTEPFVSFDRWDAILKRVGFSGVDSRTTDRDANLF<br>PTSVFSTHAIDATVEYLDAPLASSGTVKDSYPPLVVVGGQTPQSQRLLNDIKAIMPPRPLQTYKRLVDLL<br>DAEELPMKSTFVMLTELDEELFAGLTEETFEATKLLLTYASNTVWLTENAWVQHPHQASTIGMLRSIRRE

		HPDLGVHVLDVDAVETFDATFLVEQVLRLEEHTDELASSTTWTQEPEVSWCKGRPWIPRLKRDLARNNRM<br>NSSRRPIYEMIDSSRAPVALQTARDSSSYFLESAETWFVPESVQQMETKTIYVHFSCPHALRVGQLGFFY<br>LVQGHVQEGNREVPVVALAERNASIVHVRPDYIYTEADNNLSEGGGSLMVTVLAAAVLAETVISTAKCLG<br>VTDSILVLNPPSICGQMLLHAGEEIGLQVHLATTSGNRSSVSAGDAKSWLTLHARDTDWHLRRVLPRGVQ<br>ALVDLSADQSCEGLTQRMMKVLMPGCAHYRAADLFTDTVSTELHSGSRHQASLPAAYWEHVVSLARQGLP<br>SVSEGWEVMPCTQFAAHADKTRPDLSTVISWPRESDEATLPTRVRSIDAETLFAADKTYLLVGLTGDLGR<br>SLGRWMVQHGACHIVLTSRNPQVNPKWLAHVEELGGRVTVLSMDVTSQNSVEAGLAKLKDLHLPPVGGIA<br>FGPLVLQDVMLNNMELPMMEMVLNPKVEGVRILHEKFSDPTSSNPLDFFVMFSSIVAVMGNPGQANYSAA<br>NCYLQALAQQRVASGLAASTIDIGAVYGVGFVTRAELEEDFNAIRFMFDSVEEHELHTLFAEAVVAGRRA<br>VHQQEQQRKFATVLDMADLELTTGIPPLDPALKDRITFFDDPRIGNLKIPEYRGAKAGEGAAGSKGSVKE<br>QLLQATNLDQVRQIVIDGLSAKLQVTLQIPDGESVHPTIPLIDQGVDSLGAVTVGTWFSKQLYLDLPLLK<br>VLGGASITDLANEAAARLPPSSIPLVAATDGGAESTDNTSENEVSGREDTDLSAAATITEPSSADEDDTE<br>PGDEDVPRSHHPLSLGQEYSWRIQQGAEDPTVFNNTIGMFMKGSIDLKRLYKALRAVLRRHEIFRTGFAN<br>VDENGMAQLVFGQTKNKVQTIQVSDRAGAEEGYRQLVQTRYNPAAGDTLRLVDFFWGQDDHLLVVAYHRL<br>VGDGSTTENIFVEAGQLYDGTSLSPHVPQFADLAARQRAMLEDGRMEEDLAYWKKMHYRPSSIPVLPLMR<br>PLVGNSSRSDTPNFQHCGPWQQHEAVARLDPMVAFRIKERSRKHKATPMQFYLAAYQVLLARLTDSTDLT<br>VGLADTNRATVDEMAAMGFFANLLPLRFRDFRPHITFGEHLIATRDLVREALQHARVPYGVLLDQLGLEV<br>PVPTSNQPAPLFQAVFDYKQGQAESGTIGGAKITEVIATRERTPYDVVLEMSDDPTKDPLLTAKLQSSRY<br>EAHHPQAFLESYMSLLSMFSMNPALKLA
12.	R9TM73	>tr|R9TM73|R9TM73_BOTFU Polyketide synthase OS=Botryotiniafuckeliana OX=40559 GN=pks PE=4 SV=1<br>MVVPNEPIAVIGTGCRFPGGASSPSKLWNLLHHPYDLTQKVPSSRFNIKAFYHPNEALDP<br>QQRLLLETVYEALEAAGLSIEEMQGTSTAVYVGLMCADYFDVLMRDIEDIPQYLATGTAR<br>SIMSNRISYFFDWKGPSMTIDTACSSSLVAVHNAISTLRSGQSRTAIAAGANLIFGPEMY<br>IGESNLHMLSPTGRSQMWDSRADGYARGEGTAAIVLKTLKNALEDGDDIEYIIRETGVNS<br>DGKSKGITMPLAASQADLIRQTYARAGLDCTKPSERCQYFEAHGTGTPAGDPVEAEAISS<br>AFFPQRSDILNSEPLHVGSIKTVIGHLEGAAGLAGIIKAGLALKEKTIPPNLHFQSLNSA<br>IEPFYGNLNVPTAPLPWPAVEGPLRASVNSFGFGGTNAHAILESYEVCTPTPSLESTALI<br>PFTISAISEDCLIQNITNFSDYIEEHEGVNLIDLGYSLLGRSNFPTKATFVASNTEDLLD<br>QLEKVIIAKEENPNLAIGIRSTNVNDKSSRKILGVFTGQGAQWPAMGKMLIANIPSFSQT<br>IDSLEKSLRELPDAPKWSLKDEIVASVGKSSIEKAEFSQPLCTALQIALVDLLKLIGVTF<br>SAVVGHSSGEIGAAYAAGRLTAGDAIRIAYYRGLHAHLAKGKGGEEGSMMAAGLSFDEAL<br>EFCAGEEYQGKISIAASNAPKTVTLSGNKDAIEKAKSTLDDRGVFARVLKVDTAYHSDHM<br>LPCSEPYTRSLAACKISPKPSLLDCTWISSVHLKNMSSESSELETKYWVDNLVSPVRFFE<br>AVSIAAKEFGSFDAAVEVGPHPALKGPVAQTFKHAVNAVVPYTGVLSRGDNDSIAFANAL<br>GFLLNYINGKRISFKTYLDAISGGSGDHS
13.	Q6RKJ9	>tr|Q6RKJ9|Q6RKJ9_BOTFU Polyketide synthase (Fragment) OS=Botryotiniafuckeliana OX=40559 GN=PKS1 PE=4 SV=1<br>IYTRGGNFLKGDLAAFDPSFFSITAEEAKAMDPQHRLLLETTYQALENAGIPLSKASGTK<br>TGVYTGSMADDYRFTSLKDPEDLPKYAVTGTAMSLLANRLSWFFNLGGPSINLDSACSSS<br>LMALDIACQGLRNGDSSMAIVAGSCLISCLETWISLCNMGFLSPDSRCYSFDNRANGYAR<br>SEGVGVVIIKKLSDALKDNDTIRAVIRATGSNSDGHTPGITQPSREAQTRLIEETYIKGG<br>LDPAKTRYFEAHGTGTPLGDPIEAGAIGSVFREYRSATEPLYVGALKSNIGHLEGGSGIA<br>GLIKTVLVLEKGIIPPNANFEQANSNIDAEFFHLRFPTEQAPWPSQGLRRASVNSFGFGG<br>SNSHCIVNRATAKSFKFVSDPQLLVFSAVDKDGIRRVAEAYRSYFAGHALKTQRSQLSFL<br>RDLAYTLSVRRTAFVWRSFVVTDTLTDLLDLPTKISLPIRAASSLGVAFIFTGQGAQYSR<br>MGTALLSLSVFRDALKKANEIFRQLGCTWCLFSRIIFLYHLYELAHDKSASNINDPAYAQ<br>PLCTALQLALIKLLRKYGVSPTIVLGHSSGEIAAAYACGGLSFKSACKVAFFRGQVAQKI<br>RQDFKRPPRAMISINLAEFEIQPYLTKLVGSSNSGSFTTACVNSPCNVTLSGLESDIDLL<br>QQQLNTDGIFGRKLKTGVAYHSPQMNEVAHEYSHLIQSLTIGRTPLSPVVMISSVTGERI<br>ANVDCLSQAQYWVRNLVGQVNFLKAYGELVSTATSSPKRKLGAKNSSFKVCDVLEIGPHP<br>ALQRYVKDISDKLSPKPELRYHSTLSRNASNTIFIKDLAGRLSSLGYKINFDCVNEPQDP<br>CTSQQKLLVDLPEYPFNHSRSYWHEPRRQRELNLRESPELELLGTPMSDGNPLESRWRKI<br>FDPARVSWIQDHKVNGKVIYPATGMIVMAIEASKQMAPKDQKINGFRLRDIIISNPIQIS<br>SQDPKTEAQLCMRPLQNSSDKNCTSYDFRIHAINNGYWKEVCRGIIQVEYETDITEVDHG<br>KVERDEQEDYTLKHEAAARECIESISKEALYQCTSDMGVELGLSFKRLDKILYDGNKVAI<br>ADLATFDWAGQEGIDSVESHTIHPTTLDALVQLSWIPLTDGFSTKIPTMIPTRIENAWIS<br>NTGLSYQETRSIQVYCTAERKGHQKAEVSSFALDRMGELRLSLSRLESSIVSDTTTFQEK<br>LQPRQLSWYISWKPDVVFLKPHEVLRFCRSETIEEVDRIKVYQTLESMLKYFAKKALNTI<br>SEAEEKNAKPFIQKYLASLRRYLKVGEKSTCSNKLLSSAIELDDDYEIDLTIFRDMEDHP<br>LLKSYLSIGKELPGIIRGQVNPLEILFSEEKIAELYYRDICVRGTYGKDIARYLDLLAHK<br>NPGLDVLEIGSGTGSFTECILSALIGFDGTERCTERFNSYHYTDISPSFFEQAREKFGPF<br>SNRLHFRVLDIEGDLASQGFGEGSFDLVLAHSPRTYTPELSQTIETVSQKLNCVVLLLIA<br>LVQIGTEEFRSGGPCVSEETWRKVLVKNGFTGVDFSICDTDDPRYHEYSLIVSTANEEDI<br>PTTIQKLIFLVDLASSRQVEVVRNIRAYLKPSINMEIQLLSYKQTISIKDAQSSFLVFLP

<table>
<tr><td></td><td></td><td>EIEVPFLHDLSSSDFDVLKHMCISAKNILWVSHSSTGTRFAAFNGLVDGMARALRSELDI
AFVTLHIEGSGTDLKKWGETIASVLSQKLLITGMSKDMEYVAHDNLLYTGRIIEAKALDQ
QIHTRIHEQTRKVRVDQAGAVALTLKKPGLLESLQFTRDENFHLEIGPDEVEIKVQYIGL
NFRDLLVSLGRYHNNDNLLGCESSGIVTRVGVNCKTFVPGDRACMAKFGCMNTYVRAHSD
LVFQIPKGMTSEEAAGSIMPGSTAYHSLINVAKLKRGESILIHAASGATGQMAIQIAKYL
NCDIYVTVGFDKKKKILMEQYGIPEDHIFYSRNLSFVKGIRRMTQGRGIDVVLNYLSGDA
LIATWELIAPYGRFIELGTLDIYTNTKLPMSSFAKNVTFAAITIDSLNIERPKEFREIML
NVIDYFHRGIFHSVKPFHLISIGNLKEAMRLLQGGKTSGKIVLSLDSADIIPATIQPQAL
WTFDSKSTYIIAGGLGGLGRSTALWMANKGAKYLVLLSRSGPVTEAATSLLTKLSDMGVS
VEVPICDITYSDQLRKVISQFPKTFPPIKGCIQATMDILFEKMNFEEWSAATSPKVRGTW
NLHEALPDNLDFFILLSSIAGVFGSMGQSNYAAGNTFQDAFCLYRSSQGQKTISLRLGIM
SDVGIISENPDVLKIRDLATESASVKEAEFLALLDHYCSPDNPLELLGSQETLPIIGLLA
PSQFSNQELEIPSWAQTPTFSPLAYVGRDDTFSADTKPAKTNFKSQLQTAESANEVKEVV
STAIITKLTKSLGVELADIDTNKPFHAYGVDSLL</td></tr>
<tr><td>14.</td><td>Q9P855</td><td>>tr|Q9P855|Q9P855_GIBFU Polyketide synthase OS=Gibberellafujikuroi OX=5127 GN=pks4 PE=4 SV=1
MASSADVYVFGDQSTPVLDKLQALVRVKDNALLTSFLGEAFLAVRREIVSLSSLERKSIP
EAESLSLLLEGVRRSEPHAALDSAFVCIYEIGYYIDRCCHCAKDVFEISRLGVEAATVAF
RLGMHVRRRAENLGYSTPSSWSMILSSNQEELVSEALKEFSKEKNLTYSSRPYISATGPG
FTTISGPPSILESVKSCDTFSGKRLYPAPIYGPYHNSSSYSESSLEHGLASILEDVGFLE
NEMLIPIISCASGSRLDQLSFGNLLKNVLSSALSQQIRMDLVTDALVETVSGTEATLIPV
NAQTTVCSLADWLAKRGATTRIGPTLESLTKDRAEPNLAPGDENKIAIIGFSGRFPEADN
LDEFWDLLIRGLDVHKPVPEERFARDHYDPTGQRKNTSQVQYGCWLKSAGYFDTQFFHMS
PKEAMQTDPAQRLALLTAYEALEMAGVVPDRTPSTQRNRVGVYYGTTSNDWGEVNSSQDV
DTYYIPGANRAFIPGRVNYFFKFTGPSIAVDTACSSSLAAINLAITSLKNRDCNTAIAGG
TNVMTNPDNFAGLDRGHFLSRTGNCKAFNDGADGYCRADGIGTLILKRLPDAIADSDPIF
GVILGAHTNHSAESVSITRPLADAQEYLFKKLLNETGIHPHDVSYVEMHGTGTQAGDAVE
MRSVLNSFAFDHSRPRDKSLYLGSVKANVGHAESASGVLAIIKVLLMMQKNTIPPHCGIK
TKINQGFPKDLDHRGVRIALKDSVDWSRPEGGKRRVLVNNFSAAGGNTSLLLEDGPAVHP
ARQHQDGDARTEHVVAVSARSTKALEENLKALEAYIANSWAPEGELLSQLSYTTTARRVH
HSRRVAFVTNGLDDLRKSLLKAATDAGQVKGIPAVSPKVGFLFTGQGAQETAMAIGYYKS
FSSFRSDIHQLDSIATLQGLPSVLPLIHGTTPVEDLSAVVVQLGTCIIQISLARFWISLG
ITPQYVIGHSLGEYAALQIAGVLSVNDAIFLCGHRAALLDKKCTAYTHGMVAVKAAADDL
RQHISSDLKVEIACVNGAEDTVLSGPNADIESLCGKLTQAGYKLHKLEIPFAFHSSQVDP
ILDDLEELASQVGFHEPKLPIVSPLLRTLLTGDTLGPQYIRRHCRETVDFLGAIKMAESQ
GIMDRSGMCIEIGAHPILTRMVKSIIGQDFRCLASLRRKEDHFKTLADSLCALHLAGFSV
NWDEYHRDFASSRNVLQLPKYSWQLANYWMQYKYSWCLTKGDAPVENGPVGAVVQARALR
LSDSVHNVIEQVHGDKRSSITVESDMHDPSLLAIAQNHRVNGLTMAPSTLFADIAFTLAK
HLIQNHGLDTHTNLPSINNMAVEKALIVGETGPQLFRASLDMDWTTMRGSVRIFSVGANG
KQTTLHAVCDVAVENPSSHRESWQSNAYLIQRGIKQLVQGASDGSAHMMRRGLLYKIFSN
SVQYGSAFQGIEQVWFDSEGLEGTGKVFMPSGKDTFALNPYCCDSLGHITGFIMNCSDSL
DLDDHVYINHGWRTLRLVEPYQCDVQYQTYVKMQAVGSDDSTYSGDVHVLRDGKIIGICG
GVTFKKVARKVLEMLLPKPSGAKAKHGVVKHVAPEPVKHVVLTPPSTTSHSVGTTSPPEP
TESPVGSASGLIQKALEIIADEIGVDISQLTDTTLLADLGVDSLMSLTILGNFREELDLD
IPAAQFYEFSTVQDLKSFLGANDQDFSSSNSEAESSASSAASTSPSDHGDDVVEEVKPVV
AEIPRSTSTILQGTKHCSQTLFLFPDGAGSATSYVTLPSISSDMRVIGLNSPYLTKPHEF
NCALQDITGSYLNEVRRRQPQGPYHLAGWSAGGVSAFDAARQLVSEGEVVESLILIDSPN
PVGLGKLPKRMYDFLEKSGIFGAFEMGEEAQAPPDWLFQHFCVFIEALDRYVPEPFEHGM
APKTTIIWAADGVCKNPDDPRPEAQPDDPRGMNWLLNNREDFGPNGWDEFIGAGNISTMA
IENANHFTMMREPIASALCAKIRETMGVN</td></tr>
<tr><td>15.</td><td>NCBI Protein ID*: XP_044704191.1</td><td>>XP_044704191.1 fusarubin cluster-polyketide synthase [Fusarium poae]
MASHLKLYLFGDQTFEIQPHLKTLFQKRDNLFLQDFLSKAYHAIRVELFKVPYSIRKDLPRFTCLEDLLL
WDQSGQRCIALDMAMTTLYQLATFISQAGISSYDAQSTRVVGLCTGAFAAAAISCSSFTADLIPMAVYAV
VTSFRTGLLVTDTARRVDPSQNMDQSWALLVPGHKASAGVEDFCNEHDLPLTSRPYISAYAPNGITVSGP
PQRLVELAQYLSTKSITSKSIPIHGAYHAPHLYSQENARGIIANLMLNKATALSEQIPLLSGTGFKPEER
SFATLLEDAVAQVLLHPLRWTSIFEDVKASLEDTSSEKFTVVPFGTNAEHLIYTALKKTSLRSLVPDTTI
PNQKITLDSIPDSGTGKPKLAIVAMSGRFPGAKDNEAYWDLLYKGLDVHKPVPNLRWDQKTHVDPTGKNK
NTGATPFGCWLDDPGQFDARFFNISPREAPQVDPSQRLALMTAYEAIEQAGIVPDATPSTRPDRVGVFYG
VTSNDWMETNSAQNIDTYFIPGGNRAFIPGRINYFFKFSGPSYAIDTACSSSLAGIHLACNALWQNDVDT
AIAGGTNVLTNPDFHAGLDRGHFLSRTGNCKTFDDGADGYCRGEGVATIIIKRLDDALAENDPILGVVLG
AYTNHSAESESITRPHVGAQRVIFNKILNEAAVDPYTVSYVEMHGTGTQAGDATEMSSVLETFAPPVSDT
KLARANTQKLYIGSAKANIGHGEAASGVCSVVKVLQMMQKNTIVPHCGIKTKINHRFPTDFEERNVRIAF
EPTPWERISLDIPRRVMVNNFSAAGGNSALLIQDAPPKKQLTSGKDTRVQFPIAFTAKSGISLQGNLRSM
LKFLAQSPDVSLAELSYTTTARRIHHQHRVLISGSTPSEICNKIETALENNTGVNRPKSAPKMVFSFTGQ
GAQYPGMGKQLFEENTFVREELLQLDRIAQNLGFPSMLPFIQSEEQDVAKFAPSLVQLASVSLQITLSKL</td></tr>
</table>

<table>
<tr><td></td><td></td><td>WASWGIIPSAVVGHSLGEYAALNVSGVLSDTDTLFLVGGRAQLLETKCTRGTHAMLVVKGSQDEIAVALK
GVEYETACINSLIETVLAGPNDQVAKVKEILTAASFKTTLLKVPYAFHSSQLEPVVSDIEALASRVSFSK
PNIPVLCPLDGTVVQDYDIFSASYLAKHSRQPVNMLSALTTAYRDSTISDRHMMLEVGPHPAVTGMVKPT
LGQQITCIASLQRGRAPWEMLSAALKTLYDAGSSINWADYQSNFPGSHSVVALPAYSWDLKDYWIKYVND
WTLRKGDPPLVINNAPKLESTTIHRVVEEDGDSNKTHIVVEADIARKDLSPLVQGHEVDGIPLCTPSVYA
DIGLTLGKYLLEKYQPQNKDNMVVVSDMTVSKALILRGDGSKQPIQAHADADWSSQSVAIKFMSFDNKGN
LQEHSACVVRFKDRTHQETLQSQVQQTKQKMQTLRDQITTGESARFNRPMAYRMIRPLARFHDDYRAIDE
VVLNSETLEASSKISFGTVKRDGDFHTHPAVIDALTQSCGFAMNCNDHTDIDVDVYMNHGWGSLELFEAL
DFEKKYTTYTQMHAGDGGLWYGDVTIFDEDRVVAFFGQIAIQGVPRRVLKVILSIESGKKGQPQRQVQEK
PQTTSKPSTTPSSKPTQNKPAAKVEPTKFSTAIRIISEESGIEIADFTDGTTFSDVGIDSLLGLTISARF
QEELDIDLDFNALFFEHPTVKDLRNFLQGPGEDVSGSSSSAASDSGRESDTTGSATPELREEFAKSIDVE
FERALEIISEESGVARADLDDDTNFADCGVDSLLSLVIASRFQDAFDLDVRHEQLFMECQTVADLRSMLA
KEMGMVETTPTAAISEAVEVPAPVVSEAVAEETAVTHSDVSALASREKAITELVHKYSAGFSAPTSTPSA
SPLSKNGTVVLVTGASGGLGSHLVYALAQLEDVRTVICLNRTNREDPETRQYKAMRDKGIRFPENLKSKL
RIFQTDTSKPKLGLSDSEYSSMVRDVTHIIHNAWPMSAKRPLSGFESQFQVFRNLLDLGRECASGRPEDF
KFSFQMVSSIGVVGQWGLAAGQTGKIVVPEERATIDSLLGNGYAEAKWGCERMLDETLHKYPNRFRPMVV
RLGQIAGSKTSGYWNPMEHFGFLIKSSQTLNALPDVEGNLNWTPVNDIADTLTDLVLSDQAPRPIYHIDN
PVGQQWRDVNDILSDALRIPNRVPFKQWLDLVRKAPQQDNPAALLADFLEDTYLRMACGGLVLDVKHTLE
HSKSLAAVGPVSETVVRKYIHIWKEIGFLKTTAEDKAGFEAERMRLWGPRA</td></tr>
<tr><td>16.</td><td>NCBI Protein ID*: XP_014544259.1</td><td>>XP_014544259.1 Non-reducing polyketide synthase vrtA [Metarhizium brunneum]
MADVSQPRLVYFGNEFPNDDLNELFRKLLQHSKDRRFRSLSAFLDEATLVLQEEIAKLPHNVRSQVPHFD
NIVTLSENGYLRELGLGAAMESALLIVLQLALFIGHYEAKDAELNLHKDHTALAGLSVGLFSGAAVALST
SLAEVVKNGAECLRVSFRLGVHVADFSSKLEAPQPDGILESWAHVITGTSEESASEELARINKELGNPEI
AKVFISAADKASVSLSGPPSRIKAAFQHSSSLRYAKSLPLPVYDGLCHAKHLYAQEDIDAVIDGANSLVA
PSRTLQIPLISSKTGKPFLATTAGELLPEIAAELITGTIYLDNVTEGIVRHAGSVSGAEELRIDTFRTSI
VFKGILETLESSFPGREMVKNDMVSWVHRDFGTRQASNAGNAKLAIVGMACRMPGGGNDLEQFWELLEQG
RDVHTTVPPDRFDLETHYDPSGNTENAATTPYGNFIDRPGFFDAGFFTMSPREAEQTDPMQRLALTTAYE
AMEMAGLVPGRTASTRRERIGTFYGQASDDWRELNASQNIGTYAVPGGVRGFTTGRINYFFKFAGPSFCV
DTACSSSMAAVHQACMALWAGETDTAIAGGVNIITDPDNYAGLGNAHFLSKTGQCKVWDKDADGYCRAEG
IGSIVIKRLEDAEADNDNILAVVLSAATNHCADAISITHPHAGHQKDNYRRTLQKAGVNPLDVSFVEMHG
TGTQAGDAIESESVLDVFAPIKPRRRADQKLLLGALKSNIGHGEAAAGISSLIKVLLSFQKSAIPPHVGI
KTQINPTIPKDLDRRNAGLAMEMTPWPRPEGKKRIAMVNSFGAHGGNTTLLLEDPPERYRPRISPESADG
RALHPIVISARSKKSLQANLQNLLDYLDKNADVDLADLSYTTCARRMQHNLRVATAVSSVSGLQKFLRTA
IDNKAGSEAKPIPPNAPSVVLTFTGQGASYKGIRQELFDEMPYFRDQVLQLDQLVQRLGFPSVVPAITGS
DDDEVLSPVTSQLSIVVLEISLARFWAYMGVKPSAVVGHSLGEYAALAVAGVLSAADALYLVGRRAQITE
QRCHAYSHAMLSVLGTPEEIDRVLKSGAETAAIRYEVSCQNTHTDTVLGASKVDIDTIRRVLEANTFKCV
SLEIPFAYHTSQMDAVVDELEVLAQTIPFKAPSIPVLSTMLGAVVFDGKTIGAGYLRRQTRSCVKFAAAV
EAARDLGIVDDGTIWIDVGPHPVCVGFVKKIIPQARIGSSCRRNEDNISTVAKTLVTLHLAGITPNWNEY
YRANEKAYTLLNLPKYSWNETNYWIPYYGTWALDKAHLKYGGKPGNNRINGASVSSGLRTSTIHQVTHEV
IEDTRASLHVLSDIQHPEFREAVYGHTMNKCGVATSSIWTDMALAVGEYLYKKLQPQVKDVHMNVCNLEV
LHAQVASKAKGAYQPLALHADLDLDKQHMSLAWYNVNPDTSEREAEHFATAAVLFEDPASWTTEWKRTAH
LVLGRIEALQQLANENKANRVSKRMAYALFKKVVDYAEQYRGIDSMILHEYEAVADVTLAKDRHGTWHTP
PHWIDSVCHLAGLIMNGSDASNTDDYFYVTPGSDSLRFLKPLEPGGKYKSYVRMFPLPVDAGNMHAGDVY
ILQGDDIVGVLSQIRFRRVPRLLMDRFFSAPSAHNGEMISSSAARVKSTPAPVSPEAVRTPKTVVVAAEK
AVVQESDGGGLTSASSGSSDASAEIHTPPEAVEDTSVVGQCIQIIARETNLDMGELTADATFTQLGVDSL
MSLVLSEKFRNELGIDVKSSLFLECPTIGEVKAWIDQNC</td></tr>
<tr><td>17.</td><td>Q65Z23</td><td>>sp|Q65Z23|CITS_MONPU Citrinin polyketide synthase OS=Monascus purpureus OX=5098
GN=pksCT PE=1 SV=2
MIDSTSHSNLRSKAFIFGPQDLSFDVRSFNKLHSQLQNHQWVLDALASLPKLWDNFAASD
QKVQQSNTGKLLENLNAWISSGVAPEEAFPLPNVLLSPLVVIGQLVEYMTFLKAAFPDLG
KKHDLPISIKEDTETFGLCTGTLCAFAVACSSNIADIQHYGAVAARLAMLVGAIVDTEEV
LSDPEGKSVSFSASWNSAEFSDSFTHVLETFPDAYVSVIVDQRRATLTASKKTAPAIIER
LKQEGAHVTSIALSGRFHWKKHQDAVSSLIQFCGLDPDLQLADATKMLLPSRSSSDGQYI
TTGKLHELALRAILLEQSEWYKTCRISYLSKFIMDDAAVICFGPERCMPPTLARKLGPRL
TYVSEIDISSSRVPGQLLGGTQKLNLTDLPDERIAVIGMACRLPGAEDHEGFWEILKTGQ
SQHREVPEDRFGMATAWREADKRKWYGNFIDNYDTFDHKFFKKSPREMASTDPQHRLMLQ
VAYQAVEQSGYFRNNGTNRRIGCFMGVGNVDYEDNIACYPANAYSATGNLKSFLAGKISH
HFGWTGPSLTLDTACSSSSVAIHQACRSILSGECNGALAGGVNVITSPNWYHNLAGASFL
SPTGQCKPFDAKGDGYCRGEGVGAVFLKRLSSAIADGDQVFGVIASTKVYQNQNCTAITV
PNAISLSELFTDVVRQARLEPKDITLVEAHGTGTAVGDPAEYDGIRAVFGGPIRSDVLSL
GSVKGLVGHTECASGVVSLIKTLLMIQQGFIPPQASFSSINPSLNAKAEEKIEISTRLKP
WDAPFRAALINNYGASGSNASMVVTQPPNLTETPSTPLPGKSYPFWISAFDQQSLQSYVR
RLRQFLEKHAADKNLSVANLSFQVACQSNWSLPQALVFSASTKEELNRALASFEKGSTDF
PSVQLPDPKPVILCFGGQVSTYVGLDQEVYNSTAILRHYLDQCDAMCLSLGLQSIYPAIF
QRSPIEDIVQLQTALFAMQYSCAKAWIDSGLKVASVVGHSFGELIALCVSNAVSLKDAVK</td></tr>
</table>

		MISGRARLIKERWGADKGSMIAVEADLSDVEALLAKVKSQMGSETGLAIACYNASKSFTL<br>AGPTKDVDHAENLLKNDPDFSGIRYKRLNVTNAFHSVLVDALIDDLESLGQGIRFKEPTI<br>KLERATEQESTSTLNANYVATHMRKPVFFAQAVKRLSDKFPVAIWLEAGSNSTITAMASR<br>ALGTSNSSFQAVNITSEGAFRFLCDTTVKLWKEGQKVSFWAHHRLQTPMYTPVLLPPYQF<br>EKSRHWMDLKVPPKPEASVQVAEQTAIIEAPKGLTTFVGYQDASQRSVRFRVNVTTEKFN<br>RLLSGHIMANAAAVCPGMFQVEIALDALTSLRPEFQARSFIPELHDLRHYQPLVRDESRA<br>VWIEAHCPNAEGLVWNWKLTASDDKGSGSVTHTSGTITFQAADSVQVKSEFEKLRRLIGR<br>KRCLQLLDSNVADDILQGRNIYRAFTEVIDYKEIYRHVTKIAGRDNESAGRVIKTYDGET<br>WLDTVLTDCFCQVAGIFVNLMTTKIDLSERGIFICDGIDRWLRAPNAGSNNTPSQVYEVF<br>ALHHCESDSKYLSDVFAFDAREGSLVEVALGISYQKVSISGIRRVLSKGMPAGLQPQVPT<br>SPAAVAAIKTVSPPPVADSPLVDGSSTAVSGTPPTKKAPKAPSVDITGKMREIICNLSGL<br>EPDEVKDDSDLVELGIDSLMSMELAREVDLAFKTTIDVTQLIDVTDFRSLVECMQRILGI<br>DNQEDNTYLAEGLNGHEGVVTNGNAYHVNGTNGVVNGNGVLFPELGGSILPKSAILDAFR<br>IAKEATDDFILNGQLGTYYNEVMPRSTELCVAHIVNAFEQLGCPIRSAAAYQRLERVPYL<br>PKHERFMNLIYGLLEEARLIDINGSEITRTSVPVSTKSVETMLEELLHDEPLHAAEHKLT<br>SLTGSKFADCITGKEDGLQLIFGSPEGREIVTDVYAKSPINAVWIQQAEFFLEQLVKRLP<br>NTGEPLRILEMGAGTGGTTVKMLPLLERLGVPVEYTMTDLSSSLIAAARKRFKKYPFMKF<br>KVVNIESPPDPQLVHSQHIILATNCVHATRNLEISTRNIHRILRPDGFLLLLEMTEQVPW<br>VDFIFGLLEGWWLFEDGRRHALQPATHWKKILTSVGYGHVDWTEGTRPEANIQRLIIALA<br>SEPRYDHTPQSLQPPVQVPLTDIAGRQEIIDTYIREYTEDFRALPIPGIQQAVMPAPTGH<br>CVLVTGATGSLGSHVVGYLSRLPNVHTVVCLNRRSTVPATIRQEEALKVRGISLDDNSRS<br>KLVVLEVETAKPLLGLPVETYQKLVNTATHIVHSAWPMSLTRPIRGYESQFKVMQNLINL<br>AREVAAWRPVPFKFSFQFISSIGVVGYYPLRYGEIIAPEETMTADSVLPVGYAEAKLVCE<br>RMLDETLHQYPDRFRPMAVRIAQIAGSTSNGHWNPVEHFAFLIKSSQTLKALPDFDGSLS<br>WCPVDDVSATLGELLISNTTPYSIYHIENPSRQQWRKMVKTLAQSLDIPRDGIIPFDQWI<br>ERVRNSSASINDNPARQLLEFFDQHFIRMSCGNLILDTTKTREHSATLRERGPVGPGLVE<br>KYISAWKTMGFLD
18.	NCBI Protein ID*: XP_030988075.1	>XP_030988075.1 uncharacterized protein PgNI_00006 [Pyricularia grisea]<br>MSSTAKTILYFGDQTDQWIDGLDFIRKEARFAPWLQKFMADACQKLKEEMKDCESIIRDSLGDFATLYDL<br>AEMYRHTPDETGFANAFLIYIMRAAMLLRCIKREPHLLDKSESGPEWLGISGGLMSLAPLAVAKDFDTLY<br>DACVDVAGLFCRVSNFCCIRSRAVEDKSGTWGWAVLGISREDLSSVLDEYQSSVGIPPLKKAHVAVIGDR<br>WASIIGPPTVLDRCLKECPTLNGLAKNKLNIRSLQHAIPVSDIDLDYIVGNSDLVNKRLDAKHRIWGTDN<br>PDAIYGTLGEVLRAAVLQCVSRPLDIVEVVKGMAKHLDSCDQIDIKVLGTSGHTPYIATALKKQGRAVNV<br>HHIIGAPKNQISHVVQSPNGIAIVGMAGRGPGSDDLEEFWSVLMDGKDMAEEITPDRFELDEFFATEHDY<br>KDPKCTTTSKFGCFMNKPGNFDARFFRISPREAIFMDPGHRQFLMSSYEALENAGYSDGKSRNVDPGKIG<br>VFFGQSNDDWNGIAHHLKGCDAYTLQGAQRAFGAGRLAFHMGWEGPTYFLDSVCATAFSLIHFACLSLFA<br>GDVDMAVCGAANVVAYPHSWTSLSKSGILSKTGNCKPFREDADGYCRADFVGSVVLKRLGDAVAHNDNIL<br>AVIPGSGRNHSGNSPSITTSDADAQERLFNKVLANAGVLPDEISYVEMHGTGTQIGDPAEMRAVSKVFKH<br>RRSDDLVLGSIKANIGHSEAAAGMSSLLKCILMFQKGVMPPQGGMPHALNPKFPSLKQAKLEILSAAREF<br>KRQDNKPRRAMINNFDAAGGNACLLVEEYSGLPALTDGIVQVPWAEHAVVVSARTSSSLDANKRRLMGYL<br>EKNPDCRIEDVSYTTTARRIHHPIRAGYLVSSIAQLKSSLASDISSEQNKQTTAAAKPVVLVFTGQGSAH<br>GGMGGELYRTSSVFRKKVDQCVKICADHGFAQFLDIITDTDLDLSTKEAVQTQLALLTLEISLAYLWQSQ<br>AGLEPRMVMGHSLGEYAALHVAGVLSLTDMFYLVGKRAQLLEELCDPGSCTMLSVAATAETVQELIDAKP<br>DALCSISCSNAPKSTVIGGLVDDMKQLEGEMSKFGPKQLPVPYAFHTFQMDPILEPLENLARGVTFSAPK<br>FPVAPTLLAKIVDSEGIFNPSYITQQTRQQVQFVKTIKAVEEKLGGAIWLEVGPSQVCGSFVKATVNSPP<br>DSGAILSTLQKGSTSWSSFLTCVIRLYEQGAEIDWLRLYSPYREGLKLVNLPTYAWDLQEFWITHTERGR<br>QSLNTLSVPGQTHISTCAQVIVEETSSRVVIRTSMTDPGMQAIIEGHRIRDAICPGSAFCDAALTVAQRL<br>FERNERFKAKGKPVLTIRNLSLRSPLRKEPGPTSEDLVTTAMVSVQSSDDSQVTFSYRNKILGGCTVSIS<br>EGAGEESARSRTSFFIKARLDDLVAKAKQGLGHRIQSGIFYSLFASTVQYSPDFKRLKEAYISEDFTEAA<br>AEITLESNPNGTRFTSSPYHGESLVHLAGFLLNSNPSRPRSSETTFMMDNLESVQTINAAALVPGETYWT<br>FARLSSRTDDSATCDLWVFQQSQGSSELVMFCFGLRFHEVNNVVLDRLLGKNKPPAQVSEDRPKYNKSSE<br>HQVINLAPAVPSYPPQVSGPIENHDTPAAAASVKQSNGLCAAILESICRETGTDLSQLTDDTALADIGVD<br>SIMAIEITASVNKETNITLT
19.	NCBI Protein ID*: XP_029743834.1	>XP_029743834.1 uncharacterized protein PpBr36_10843 [Pyriculariapennisetigena]<br>MPRAPVLCITHGGGPMPVLEPQMGGHGHDPLNESLRTRVRDILKLGTAEAPRAIVVITPHLRPDYPMVTM<br>VDKPPICHDFEPSHPPAAWEIQYEAPGSSEVSNLVYDALSSAGLNPAKDFERGFDHGVFIPFIFISPKGE<br>VPLISVTISSSSDPELYTRMGRALIKLREDNVAVVGSGFASYHNVPMMRKLYMADAASPEMIEWRDKVAK<br>FNKGLINAVTTQDPGRRAELLRSWRDLPHSYDVHPDNEDEHILPLFVAAGAADSSKGDYAESSPEILTPR<br>ESVCVAAACGGLLNAVALMYARDMASLCQAGLDTIRMARRLSQIVLWRARSLHNGPGCWGWLVSGIKAGD<br>LQAMLDSYHAVESTPNHRTIKVAGSGIADAWHTVIGPPAVLEDFFGQYPPCQRLTKSRLQVTGPVHVIPD<br>VQLAEVEHIAAGMTSVHGMFDSSPRTDIQLVSLGEGGLLSAPTWKELLDLVVREILSQPLNMTRGLSAVE<br>SAIPHEATVDIFGAGPSPHIPTLCVYLKSKGRKVVSVATEPKSPSAPLPGQSSGRIAIVGMGGRYPRSGD<br>LEEFWDKICQGKALHKERFSLDDYYSSSNTPHTTNTKYGCFLDSPGQFDARFFNLSPREAMEVDPSHRLF<br>LLAVYEALEVAGYFRRPHTKTESCRFATIVGQISDDWTEIIRLRGGDAFSLTGNQRSFAAGRVNYHFKWA<br>GPAVTLDTACSSSMTALTQACTLLLTGDCDMAVAGGTNIFSSPFNFNILGKAGFTSTTGGCKSYRADADG

<table>
<tr><td></td><td></td><td>YCRGEFVGAFVLKRYEDALKDNDNILATVLGSARNHSGNAISITHSDHEAQEHLMAEVLRKAHLEASDVS
YVEMHGTGTQVGDYAEMKAVSNALGRNRRKDRLRVGSIKANAGHGEAGAGAAAVIKAIMMFEKNIIPPQA
GLPAKLNPRFPPLSELNIDIPAGVTEFKSAPERPRRILINNFDASGGNTCLLLEEPPARKPVASIPLPSR
HVVVTSAKSAKSHESNKRNLLAYIKQNPAVQIQDLAYTTTARRIHHPIRSAYAATSVAELERQIEMDLAS
TPASPEINSSATRSPVVFMFSGQGQLYAGMGADLYRDSPAFRDEMDKCVRICASFGFPSFEDLVKDPAAT
LAGRPTSETQLATVCLEISLAAFWASCGLVPAVVMGHSLGEFAALHVAGVLTLADTLLLVGKRALVMEAR
CQAGIFSMLQLAAPLSVVQELLRNHADISISCINGPAAIVVSGPAGDVAFLQTSCRTQQIRFTLLKLPYA
FHSHQMDPALDEYEAVARTAHFDAPKVPIASTLLGHLVSEAGQVDAKYMVRQTREPVDFVGAVKVVEAKL
KDCLWLELGPSSVLVDLVKPMLQPSGQARTGCSLKSGTSAWTSISAALAAAYEAQQEIDWAGFHAPYISG
LRLLRLPFYAFDLKNYWRSYPARVDPVSRLAAPSQSHGLPPPPLSTCLHYVVSESGSTSTFRSHLAEPEL
KALIEGHRLMDVSISPACVLGEMAMAATTHMLKKVGREVKAIQLGARDCVFFSPVSIDASRADQSVVITA
ELDRAGDVVDVKMLADTGAVAAQLKVQLVNPMQHQQQWASFSPFIWQCCSAVVEAARSGRGQRLTPAFFY
SIFAQTVSYSSAFQRIVDVYLSPDMGEGVAEVVLGPTPDSCTFTLNPYWVDSLTHLAGFLVNGDPAKPRG
TMFMMNTYDSFDIVEPLEPGVAYTVYARIVRKEKKSSVVNVYVFREQRLVMQNLGLHLQQIPTSLIASMI
GKTSQDGASAATKQVGPLARSDPARTPMRQHPAAPALIETQAQPPQGPSQKAQQESSSSSSSSSSSSIAD
LLMSTIAEETGCEISDLTPNTILADLGVDSIMAIQIAEKLQAKLGCEIGAALFHQHPSISELQAALGSRS
APANVVQNQETAVPAKAVSASLRLQTQLNREFHNHDLSEVLISTIARETGAEQSELEVPHTSLADLGVDS
IMAMQIASSFLEQTGVDLHPSALVENPTMSDLSRMFQQLQQHPEPESSSTSGFVTVEHDQSLEESVLASA
SDPGPELVVDSTPVDALHALLAPAAPPKDACKPKVNAVLMHGRKTSGLAPLFMFPDGAGGASTYMYLKRF
RNDRPMYVLESPYLHNPEAFTVGVETVAAMFKQVVLDIYPAGGFLLSGYSGGAVYAYETARQLMADGHVV
QGLLLFDMAVPQLRPDPGMPPAMTLLLPQKLVKAREWADPAVMRGQLAHMAQMVRTVSEYDAVPMPADRR
PRRTWITWCKRGVVERLDDLVINQLRKKGILVDSIPNFMEDPAVGPFAWAIPKGKPLGPNGWDRLVGNVR
CSSIDADHFTMVIPPDVAKFQKALEDALVYCTED</td></tr>
<tr><td>20.</td><td>NCBI Protein ID*: G0R6S9.1</td><td>>sp|G0R6S9.1|SORB_HYPJQ RecName: Full=Non-reducing polyketide synthase sor2; Short=NR-PKS sor2; AltName: Full=Sorbicillinoid biosynthetic cluster protein 2
MAASSTRTLLMFGPGAMSLNETYFASILSFISTDSASQWALSAVRDIESHWPSLCEAIPKLQHTSGVSNA
QKLAEWLRTGTLAPGSTIASLPNAILGPLVVIAQLVEYLRHVDSLSESGLRGGEGFQVPSAPDAETVGCC
LGTFSALVVSSSSSWAQFCHNASAVVRIVFVLGALSDAQDATDASGPSVSLIAFWRGGQSLSDLKKALEK
FPEAYISVLYDENRVTVTTSTRTVAALKNHLQTVGITTNETEFHGRFHAAQLYQTELEAFLAYCRRFPTF
QLPDSSCIVVPTRVNSENVVTSQESLLEISCRAFLVSQFDWIKTFRAAVSSTLQNRASRIIEFGPERCVP
PTLLRRLNSQVTHFDFEESIKRAKASLSHDQELPAGVAENDIAVIGMACKVAGADDVNEYWELLLQGKSQ
HRELIPNDRFVMETPFRPFEAGDDKKKWFGNFIGDHDAFDYKFFKKSPREALHMDPQQRLMLQAAYQAVA
QSGYYNANLNVHGPTKVGCYIGVVANDYENNISHTTPIAFSATGALRSYIAGKVSHFFGWTGPAMTVDTA
CSASTVALDLACKAILSGECSAALVGGTNFFSTPMFFQNLAAGSFLSTTGQCKPFDAKADGYCRGEAVGT
VFLKKLSQAIADGDQVLGVISATAINQNRNETPIFVPNSPSLTNVFRTAIEKSGLDAKDISVVEAHGTGT
PVGDPVEYDSIRQVFGGAVRAGQDALQVGSVKGLVGHTEGASGVVALVKILLMLQRGQIPPQASFETINP
SIKYSPSDNLEITKTPLPWNQEFKAALINNYGAAGSNASVIIKQGPAQLLRRLPPVVDSETEALLSKVGA
DDAQKAPFFISGLDEKAILAYAQKLRQFIYSHSNLDIQDLAFSVNRQSNWSLGRGAVFSAGSIAELDEKL
ASIETFPVPSSQPPVILCFGGQVSTFVGLDYQLFAKSAILRRHLDQCDAACKSIGAGSIYPRIFQSDPID
DPSVLQPLLFSLQYSCAMSWIDCGIVPAKLVGHSFGELTALCISGVVSLQDGLKLVYGRSKIIKESWGAE
RGAMLAVEADLEELESLLTTVNSSLQEGRATIACFNGPRSFTVAGSSAAIDAVQQAISNTQPVLKHKRLN
VTNAFHSVLVERLKPDLEALGRQLTFALPQIPLERATRSREDHGLSPSYVANHMREPVYFHHAVERIAKE
YPEAIWLEAGSNSTITTMASKALGLPKASTFQPVNVTNNSKASSQLSDVTMNLWKAGLRITFWPHSRAQT
YEYKHIILPPYQFEKHRQWLEFKPPQALQVVVQTDSSTDARNGTTEPQPVGLYTLLDRGQDKYRFRVNTA
AGQFVDAMSDHAIGKAQTLPAMFGVDLAIEALLSIHPELGDTSRFDPQIYNVVNQEYIHDSSRALFVLFE
RLGQDENSWAFELTSKGKDGAESLHMSGQLHFQASDDARSRLEFSRLDRFITHDRCLQVLESSGRSDEVI
QGQTIYSVLSSSDVNYGQRLRGLQRLVGRSNESAGRLVRRRSGKLFVDFALGEVFTQVGSIWANCMAQHQ
RNTRNKGIYMATGLEQWSKSPKVLQKFNQGLYDNDPEIEWHVLAQHKRNTSDDSFTTDIFVFDAASGDLE
EVILGIKYAPVSLDQLFSGSAIATATTPVANGYVPLTTPFVPVPTTTKQAAVPQPQHVAKKAAPRAAPKR
DIKEELWLRLRPVLADISGLEPEEIQPTDALADIGIDSLMGMEMAREVETTFNCTVEQSELLSIVDVPGI
LKFLQSTLGDEDVHDSSETMSTVSSDGNVHSPPTSGSEMASPNLKVSYGSAYGSSDLPISAVLEAFGESS
AKTDQFLKTYGCAGYLDGVSQKQTRLCLVLTSAAFKQLGCDLEAAKPGEVLQPVPFVERHRRFHQYLYSM
LEETRIINIDGDVITRTAIPLPSQSADAILQDLMRRHADNGSSHQLAYNVGSRMADVLSGKADGPQLIFG
DAKNRELVANFYGELPFNKLYFELMADFLTRLANKLKLSSSRSGTPTLKILEMGAGTGGTTKVLLPILAK
LGIPLEYTFTDLSPSLVAQAKRRFKEYPFMKFAVHDIEQPPSDPELVGSQHVVIASNAVHATHSLRDSAR
NIHKFLRPDGFLMLLEMMRTLHWVDVVWGTLEGWWLFDDGRTHAIVNEQRWEKELLASGFKHVRWTDGKL
PEVHVQRVIIALAGDGDGDVSDIPALTSPALKDEEDHGSKLDGEERKRVANAYVESTIRDFAIPSYTGPI
LSTAPGAGACVLVTGATGSLGSHLVAHIAGLPSVDTIYCLNRPRPGKKGQEDQSRDPLSRLTEVLASKSI
QLSEAEISKLRVIETDLPLPQFGLDEIQYEQLLNNVTHIVHSAFPVNGLRSLKQNEPQFTLMRNLVDLAA
GVSARRPTEFKFTFQFISSLSAVGMYPKVHGEKRVPEQQWDVDSALPNGYGEAKVICERVLLETLGQHPD
RFRAMTVRLGQLSGSMKTGYWNHMEMLSFLFKSAQTLRALPDVDGDVSWLHLEDASASLADLLLRDAPTC
HAVYHLDNPRPRDWKDVIPVLADALDIPSSHIVPFEEWLRRVRAYPGEDPWDNPSAKAMDFFEHKFKHMS
CGGVTMATDHALEHSETLRAVQPVSDELVRKYIQAWKDSGFLR</td></tr>
</table>

Table S3. Identification of the protein domains present in Interactive Type 1 NR-PKS

Sl. No.	iNR-PKS	Domain (Pfam ID)/ Position (amino acid positions)								
		ACP transacylase **(SAT)**	Ketosynthase **(KS)**	Acyltransferase **(AT)**	Product Template **(PT)**	Acyl Carrier Protein **(ACP)**	Thioesterase **(TE)**	Ketoreductase **(KR)**	Dehydratase **(DH)**	Enoylreductase **(ER)**
		**(PF16073)**	**(PF00109)**	**(PF00698)**	**(PF08017)**	**(PF00550)**	**(PF00975)**	**(PF08659)**	**(PF08242)**	**(PF00106)**
1.	StcA (XP_050468055.1)	1-200	250-550	600-900	950-1150	1200-1300	1500-1700	-	-	-
2.	PkgA (Q5AXA9)	1-180	200-500	550-850	900-1100	1150 -1250	1400-1600	-	-	-
3.	AptA (Q5B0D0)	12-182	203-503	553-851	897-1102	1123-1225	1435-1198	-	-	-
4.	Pks13(G3KLH6.1)	1-210	250-550	600-950	-	1000-1100	1500-1700	-	-	-
5.	VrtA (XP_014544259.1)	1-185	210-520	550-880	910-1125	1150-1255	1450-1620	-	-	-
6.	WA(Q03149)	1-498	220-530	560-890	925-1140	1165-1270	1480-1655	-	-	-
	PksP(Q4WZA8)	1-192	215-525	55-885	-	920-1025	1250-1425	-	-	-
7.	MdpG (XP_657754.1)	1-178	205-515	545-875	910-1120	1145-1250	1380-1555	-	-	-
8.	OrsA (Q5AUX1)	1-184	210-520	550-880	-	900-1005	1100-1275	-	-	-
9.	Pks1 (L7XAV2.1)	1-188	215-525	550-880	-	900-1005	1100-1275	-	-	-
10.	HypS (XP_030988075.1)	-	~50–450	~500–750	-	~800–850	~900–1000	-	-	-
11.	RadS (XP_029743834.1)	-	~50–430	~450–700	-	~720–780	~800–950	-	-	-
12.	Sor2 (G0R6S9.1)	-	~50–420	~450–720	-	~740–800	~820–950	-	-	-
13.	AfoE (A0A5N6H990)	5-150	180-500	520-780	-	800-860	880-1020	-	-	-
14.	PksCT(Q65Z23)	1-142	165-492	510-780	795-925	940-1000	1015-1150	-	-	-
15.	Bik1(Q9P855)	-	~45-420	~450-710	-	~725-785	~800-950	~650-740	~530-620	-
16.	Fsr1 (XP_044704191.1)	1-148	165-495	510-780	795-920	935-995	1010-1145	-	-	-

Table S4. Structural validation of the protein 3D models

Cluster: Chemical Nature of BGC (KO No.)	Name of the Representative iT1-NR-PKS	Template Used	Sequence Similarity	Coverage	Sequence Identity coverage	GMQE	MolProbity Score	Clash Score	Ramachandran Favoured
**Cluster 1:**Noranthrone (K15316)	StcA	Q12397.1.A	0.61	1.00	100.00%	0.82	1.66	1.32	90.86%
**Cluster 2:**Asparasone (NA)	Fsr1	S0DTP6.1.A	0.57	1.00	84.98%	0.84	1.19	0.54	93.88%
**Cluster 3:**Naphtho-gamma-pyrone (K15321)	WA	Q03149.1.A	0.61	1.00	100.00%	0.85	1.31	0.39	92.34%
**Cluster 4:**Citrinin (NA)	PksCT	Q65Z23.1.A	0.61	1.00	100.00%	0.84	1.05	0.35	95.06%
**Cluster 5:** Zearalenone (K15417)	Pks1	L7XAV2.1.A	0.61	1.00	100.00%	0.85	1.10	0.44	93.75%
**Cluster 6:**Orsellinic acid (K15416)	OrsA	Q5AUX1.1.A	0.61	1.00	100.00%	0.83	1.34	0.66	93.34%
**Cluster 7:** Asperthecin (K15317)	Pks13	G3KLH6.1.A	0.61	1.00	100.00%	0.88	1.11	0.33	94.42%
**Cluster 8:**Monodictyphenone(K15415)	MdpG	Q5BH30.1.A	0.61	1.00	100.00%	0.89	1.06	0.40	94.84%
**Cluster 9:** Bikaverin (NA)	Bik1	S0DZM7.1.A	0.61	1.00	99.70%	0.86	1.32	0.72	93.97%

Table S5: Distribution of iT1-NR-PKS across Ascomycota obtained from KEGG Genome database

Sl. No.	Name of the Fungi (Ascomycota)	Class	Order	Family	NR-PKS Type	Locus Tag	Cluster
1.	*Fusarium graminearum* (Gibberella zeae PH-1)	Sordariomycetes	Hypocreales	Nectriaceae	Zearalenone synthase	FGSG_02395	Cluster 5
2.	*Fusarium pseudograminearum* CS3096	Sordariomycetes	Hypocreales	Nectriaceae	Zearalenone synthase	FPSE_06864	Cluster 5
3.	*Fusarium falciforme* Fu3.1	Sordariomycetes	Hypocreales	Nectriaceae	Naphtho-gamma-pyrone polyketide synthase	NCS54_00237700	Cluster 3
4.	*Fusarium poae* DAOMC 252244	Sordariomycetes	Hypocreales	Nectriaceae	Naphtho-gamma-pyrone polyketide synthase	FPOAC1_002477<br>FPOAC1_012524	Cluster 3
5.	*Fusarium musae* F31	Sordariomycetes	Hypocreales	Nectriaceae	Naphtho-gamma-pyrone polyketide synthase	J7337_003707	Cluster 3
6.	*Fusarium venenatum* A3/5	Sordariomycetes	Hypocreales	Nectriaceae	Naphtho-gamma-pyrone polyketide synthase	FVRRES_13609<br>FVRRES_02715	Cluster 3
7.	*Fusarium keratoplasticum*	Sordariomycetes	Hypocreales	Nectriaceae	Naphtho-gamma-pyrone polyketide synthase	NCS57_00233100<br>NCS57_01209000	Cluster 3
8.	*Trichoderma reesei* QM6a	Sordariomycetes	Hypocreales	Hypocreaceae	Asperthecin polyketide synthase	TRIREDRAFT_81964	Cluster 7
9.	*Trichoderma reesei* RUT C-30	Sordariomycetes	Hypocreales	Hypocreaceae	Asperthecin polyketide synthase	M419DRAFT_90904	Cluster 7
10.	*Trichoderma atroviride* P1	Sordariomycetes	Hypocreales	Hypocreaceae	Monodictyphenone polyketide synthase	TrAtP1_013134	Cluster 8
					Naphtho-gamma-pyrone polyketide synthase	TrAtP1_010560	Cluster 3
11.	*Trichoderma asperellum* FT101	Sordariomycetes	Hypocreales	Hypocreaceae	Monodictyphenone polyketide synthase	TrAFT101_006398<br>TrAFT101_009627	Cluster 8
					Naphtho-gamma-pyrone polyketide synthase	TrAFT101_010755	Cluster 3

12.	*Metarhizium acridum*CQMa 102	Sordariomycetes	Hypocreales	Clavicipitaceae	Asperthecin polyketide synthase	J3458_020328	Cluster 7
13.	*Metarhizium robertsii* ARSEF 23	Sordariomycetes	Hypocreales	Clavicipitaceae	Asperthecin polyketide synthase	MAA_08920	Cluster 7
14.	*Metarhizium brunneum* 4556	Sordariomycetes	Hypocreales	Clavicipitaceae	Asperthecin polyketide synthase	G6M90_00g056690	Cluster 7
					Naphtho-gamma-pyrone polyketide synthase	G6M90_00g031450<br>G6M90_00g069690<br>G6M90_00g079000<br>G6M90_00g113840	Cluster 3
					Noranthrone synthase	G6M90_00g105870	Cluster 1
15.	*Pochoniachlamydosporia* 170	Sordariomycetes	Hypocreales	Clavicipitaceae	Monodictyphenone polyketide synthase	VFPPC_02813	Cluster 8
					Zearalenone synthase	VFPPC_08964	Cluster 5
					Naphtho-gamma-pyrone polyketide synthase	VFPPC_10343<br>VFPPC_11725	Cluster 3
16.	*Thermothelomyces thermophilus* ATCC 42464	Sordariomycetes	Sordariales	Chaetomiaceae	Monodictyphenone polyketide synthase	MYCTH_101261	Cluster 8
17.	*Podosporapseudoanserina* CBS 124.78	Sordariomycetes	Sordariales	Podosporaceae	Noranthrone synthase	QC764_200510	Cluster 1
18.	*Pyriculariapennisetigena* Br36	Sordariomycetes	Magnaporthales	Pyriculariaceae	Zearalenone synthase	PpBr36_10843	Cluster 5
19.	*Pyricularia grisea* NI907	Sordariomycetes	Magnaporthales	Pyriculariaceae	Zearalenone synthase	PgNI_00006<br>PgNI_05168	Cluster 5
20.	*Pestalotiopsis fici* W106-1	Sordariomycetes	Xylariales	Sporocadaceae	Monodictyphenone polyketide synthase	PFICI_10824	Cluster 8
22.	*Aspergillus nidulans* FGSC A4	Eurotiomycetes	Eurotiales	Aspergillaceae	Naphtho-gamma-pyrone polyketide synthase	ANIA_08209	Cluster 3
					Noranthrone synthase	ANIA_07825	Cluster 1
					Monodictyphenone polyketide synthase	ANIA_00150	Cluster 8
					Asperthecin polyketide synthase	ANIA_06000<br>ANIA_07071	Cluster 7
					Orsellinic acid synthase	ANIA_07909	Cluster 6

22.	*Aspergillus fumigatus* Af293	Eurotiomycetes	Eurotiales	Aspergillaceae	Asperthecin polyketide synthase	AFUA_7G00160	Cluster 7
					Monodictyphenone polyketide synthase	AFUA_4G14560	Cluster 8
					Naphtho-gamma-pyrone polyketide synthase	AFUA_2G17600	Cluster 3
23.	*Aspergillus oryzae* RIB40	Eurotiomycetes	Eurotiales	Aspergillaceae	Noranthrone synthase	AO090026000009	Cluster 1
					Naphtho-gamma-pyrone polyketide synthase	AO090102000545	Cluster 3
					Asperthecin polyketide synthase	AO090701000530	Cluster 7
24.	*Aspergillus niger* CBS 513.88	Eurotiomycetes	Eurotiales	Aspergillaceae	Asperthecin polyketide synthase	An11g07310	Cluster 7
					Naphtho-gamma-pyrone polyketide synthase	An09g05730	Cluster 3
25.	*Aspergillus fischeri* NRRL 181	Eurotiomycetes	Eurotiales	Aspergillaceae	Naphtho-gamma-pyrone polyketide synthase	NFIA_093000	Cluster 3
					Asperthecin polyketide synthase	NFIA_112240	Cluster 7
					Monodictyphenone polyketide synthase	NFIA_101660 NFIA_101810	Cluster 8
26.	*Aspergillus flavus* NRRL3357	Eurotiomycetes	Eurotiales	Aspergillaceae	Asperthecin polyketide synthase	AFLA_005055	Cluster 7
					Naphtho-gamma-pyrone polyketide synthase	AFLA_011972	Cluster 3
27.	*Aspergillus clavatus* NRRL 1	Eurotiomycetes	Eurotiales	Aspergillaceae	Naphtho-gamma-pyrone polyketide synthase	ACLA_076490	Cluster 3
28.	*Aspergillus luchuensis* IFO 4308	Eurotiomycetes	Eurotiales	Aspergillaceae	Naphtho-gamma-pyrone polyketide synthase	AKAW2_40582A AKAW2_50916A	Cluster 3
					Asperthecin polyketide synthase	AKAW2_80648A	Cluster 7

29.	*Aspergillus chevalieri* M1	Eurotiomycetes	Eurotiales	Aspergillaceae	Monodictyphenone polyketide synthase	ACHE_20978A	Cluster 8
					Naphtho-gamma-pyrone polyketide synthase	ACHE_40803A<br>ACHE_50703S	Cluster 3
30.	*Aspergillus puulaauensis* MK2	Eurotiomycetes	Eurotiales	Aspergillaceae	Asperthecin polyketide synthase	APUU_11045A	Cluster 7
					Monodictyphenone polyketide synthase	APUU_11999A	Cluster 8
					Naphtho-gamma-pyrone polyketide synthase	APUU_60653S	Cluster 3
31.	*Penicillium rubens* Wisconsin IBT 27055	Eurotiomycetes	Eurotiales	Aspergillaceae	Naphtho-gamma-pyrone polyketide synthase	N7525_008132	Cluster 3
32.	*Penicillium digitatum* Pd1	Eurotiomycetes	Eurotiales	Aspergillaceae	Naphtho-gamma-pyrone polyketide synthase	PDIP_48950	Cluster 3
33.	*Penicillium oxalicum* HP7-1	Eurotiomycetes	Eurotiales	Aspergillaceae	Naphtho-gamma-pyrone polyketide synthase	POX_a00499	Cluster 3
					Monodictyphenone polyketide synthase	POX_a00753	Cluster 8
34.	*Talaromycesmarneffei*	Eurotiomycetes	Eurotiales	Trichocomaceae	Monodictyphenone polyketide synthase	EYB26_001619<br>EYB26_006526	Cluster 8
					Naphtho-gamma-pyrone polyketide synthase	EYB26_002530<br>EYB26_004255	Cluster 3
35.	*Talaromycesrugulosus* W13939	Eurotiomycetes	Eurotiales	Trichocomaceae	Asperthecin polyketide synthase	TRUGW13939_00034	Cluster 7
					Naphtho-gamma-pyrone polyketide synthase	TRUGW13939_08432	Cluster 3
36.	*Coccidioides immitis* RS	Eurotiomycetes	Onygenales	Onygenaceae	Noranthrone synthase	CIMG_08569	Cluster 1
37.	*Trichophyton benhamiae* CBS 112371	Eurotiomycetes	Onygenales	Arthrodermataceae	Asperthecin polyketide synthase	ARB_00538	Cluster 7

38.	*Cercospora beticola* 09-40	Dothideomycetes	Capnodiales	Mycosphaerellaceae	Naphtho-gamma-pyrone polyketide synthase	CB0940_00833<br>CB0940_10910	Cluster 3
					Monodictyphenone polyketide synthase	CB0940_02338	Cluster 8
					Noranthrone synthase	CB0940_02994	Cluster 1
39.	*Pyrenophora tritici-repentis* M4	Dothideomycetes	Pleosporales	Pleosporaceae	Zearalenone synthase	PtrM4_088550	Cluster 5
40.	*Alternaria dauci* A2016	Dothideomycetes	Pleosporales	Pleosporaceae	Monodictyphenone polyketide synthase	ACET3X_000232	Cluster 8
					Asperthecin polyketide synthase	ACET3X_002037	Cluster 7
					Zearalenone synthase	ACET3X_007864	Cluster 5

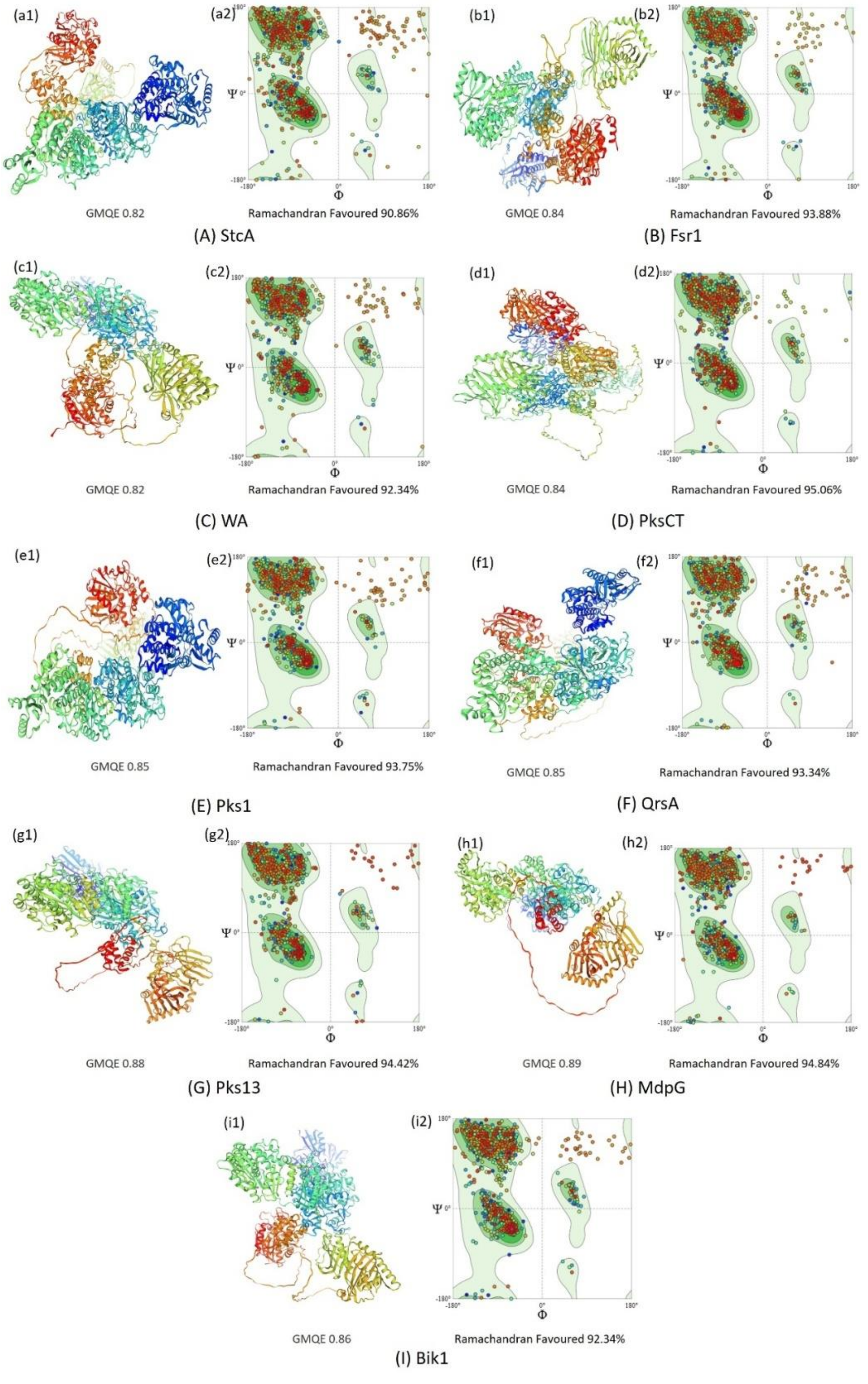

Figure S1. Tertiary structure and Ramchandran analysis: A. StcA (a1. 3D structure, a2. Ramchandran plot); B. Fsr1 (b1. 3D structure, b2. Ramchandran plot); C. WA (c1. 3D structure, c2. Ramchandran plot); D. PksCT (d1. 3D structure, d2. Ramchandran plot); E. Pks1 (e1. 3D structure, e2. Ramchandran plot); F. OrsA (f1. 3D structure, f2. Ramchandran plot); G. Pks13 (g1. 3D structure, g2. Ramchandran plot); H. MdpG (h1. 3D structure, h2. Ramchandran plot); and I. Bik1 (i1. 3D structure, i2. Ramchandran plot).

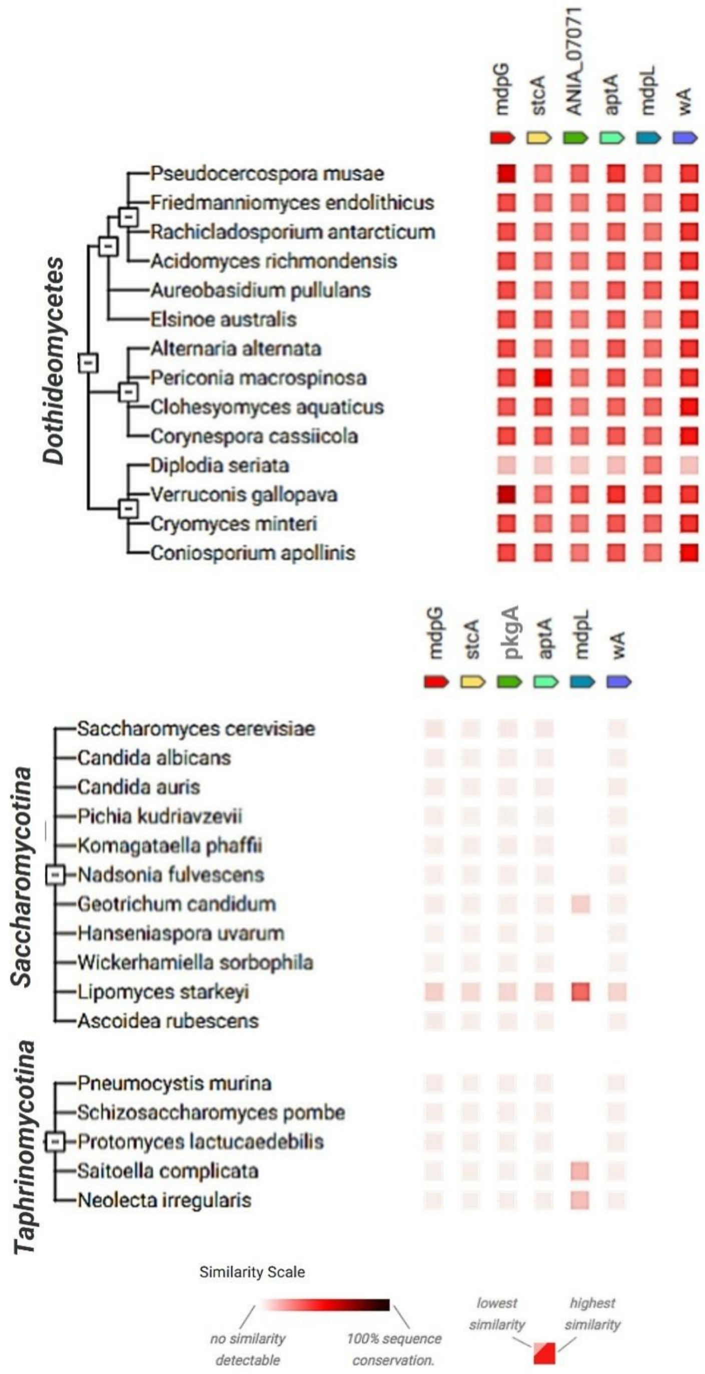

Figure S2. Distribution of different iT1-NR-PKS across the fungi phylum Ascomycota:Phylogenetic tree showing co-expression of wA, stcA, mdpG, aptA, mdpL, and orsA across the class Dothideomycetesof subphylum Pezizomycotina, subphylumSaccharomycotina and subphylumTaphrinomycotina.

References

41. Kjærbølling, I.; Vesth, T.C.; Nybo, J.L.; Theobald, S.; Frisvad, J.C.; Kogle, M.E.; Lyhne, E.K.; Kuo, A.; Salamov, A.; Riley, R. Friends and foes-comparative genomics of 23 *Aspergillus Flavi* species. In Proceedings of the 14th European Conference on Fungal Genetics, 2018.

42. Nierman, W.C.; Pain, A.; Anderson, M.J.; Wortman, J.R.; Kim, H.S.; Arroyo, J.; Berriman, M.; Abe, K.; Archer, D.B.; Bermejo, C. Genomic sequence of the pathogenic and allergenic filamentous fungus *Aspergillus fumigatus*. *Nature* **2005,** 438, 1151-1156.

43. Galagan, J.E.; Calvo, S.E.; Cuomo, C.; Ma, L.-J.; Wortman, J.R.; Batzoglou, S.; Lee, S.-I.; Baştürkmen, M.; Spevak, C.C.; Clutterbuck, J. Sequencing of *Aspergillus nidulans* and comparative analysis with A. fumigatus and *A. oryzae*. *Nature* **2005,** 438, 1105-1115.

44. Mayorga, M.E.; Timberlake, W.E. The developmentally regulated *Aspergillus nidulans* wA gene encodes a polypeptide homologous to polyketide and fatty acid synthases. *Mol. Gen. Genet*. **1992,** 235, 205-212.

45. Wortman, J.R.; Gilsenan, J.M.; Joardar, V.; Deegan, J.; Clutterbuck, J.; Andersen, M.R.; Archer, D.; Bencina, M.; Braus, G.; Coutinho, P. The 2008 update of the *Aspergillus nidulans* genome annotation: a community effort. *Fungal Genet. Biol*. **2009,** 46, S2-S13.

46. Li, Y.; Chooi, Y.-H.; Sheng, Y.; Valentine, J.S.; Tang, Y. Comparative characterization of fungal anthracenone and naphthacenedione biosynthetic pathways reveals an α-hydroxylation-dependent Claisen-like cyclization catalyzed by a dimanganese thioesterase. *J. Am. Chem. Soc*. **2011,** 133, 15773-15785.

47. Xu, Y.; Espinosa-Artiles, P.; Schubert, V.; Xu, Y.-m.; Zhang, W.; Lin, M.; Gunatilaka, A.L.; Sï¿ ½ssmuth, R.; Molnï¿ ½r, I.n. Characterization of the biosynthetic genes for 10, 11-dehydrocurvularin, a heat shock response-modulating anticancer fungal polyketide from *Aspergillus terreus*. *Appl. Environ. Microbiol*. **2013,** 79, 2038-2047.

48. Hendrickson, L.; Davis, C.R.; Roach, C.; Nguyen, D.K.; Aldrich, T.; McAda, P.C.; Reeves, C.D. Lovastatin biosynthesis in *Aspergillus terreus*: characterization of blocked mutants, enzyme activities and a multifunctional polyketide synthase gene. *Chem. Biol*. **1999,** 6, 429-439.

49. Hu, Y.; Hao, X.; Lou, J.; Zhang, P.; Pan, J.; Zhu, X. A PKS gene, pks-1, is involved in chaetoglobosin biosynthesis, pigmentation and sporulation in *Chaetomium globosum*. *Sci. China Life Sci*. **2012,** 55, 1100-1108.

50. Kroken, S.; Glass, N.L.; Taylor, J.W.; Yoder, O.; Turgeon, B.G. Phylogenomic analysis of type I polyketide synthase genes in pathogenic and saprobic ascomycetes. *Proc. Natl. Acad. Sci. U.S.A*. **2003,** 100, 15670-15675.

51. Linnemannstöns, P.; Schulte, J.; del Mar Prado, M.; Proctor, R.H.; Avalos, J.; Tudzynski, B. The polyketide synthase gene pks4 from *Gibberella fujikuroi* encodes a key enzyme in the biosynthesis of the red pigment bikaverin. *Fungal Genet. Biol*. **2002,** 37, 134-148.

52. Witte, T.E.; Harris, L.J.; Nguyen, H.D.; Hermans, A.; Johnston, A.; Sproule, A.; Dettman, J.R.; Boddy, C.N.; Overy, D.P. Apicidin biosynthesis is linked to accessory chromosomes in *Fusarium poae* isolates. *BMC Genomics* **2021,** 22, 591.

43. Saud, Z.; Kortsinoglou, A.M.; Kouvelis, V.N.; Butt, T.M. Telomere length de novo assembly of all 7 chromosomes and mitogenome sequencing of the model entomopathogenic fungus, *Metarhizium brunneum*, by means of a novel assembly pipeline. *BMC Genomics* **2021,** 22, 87.

54. Shimizu, T.; Kinoshita, H.; Ishihara, S.; Sakai, K.; Nagai, S.; Nihira, T. Polyketide synthase gene responsible for citrinin biosynthesis in *Monascus purpureus*. *Appl. Environ. Microbiol*. **2005,** 71, 3453-3457.

55. Gómez Luciano, L.B.; Tsai, I.J.; Chuma, I.; Tosa, Y.; Chen, Y.-H.; Li, J.-Y.; Li, M.-Y.; Lu, M.-Y.J.; Nakayashiki, H.; Li, W.-H. Blast fungal genomes show frequent chromosomal changes, gene gains and losses, and effector gene turnover. *Mol. Biol. Evol*. **2019,** 36, 1148-1161.

56. Martinez, D.; Berka, R.M.; Henrissat, B.; Saloheimo, M.; Arvas, M.; Baker, S.E.; Chapman, J.; Chertkov, O.; Coutinho, P.M.; Cullen, D. Genome sequencing and analysis of the biomass-degrading fungus *Trichoderma reesei* (syn. *Hypocrea jecorina*). *Nat. Biotechnol*. **2008,** 26, 553-560.
